# Role of the Meso Substituent in Defining the Reduction of Uranyl Dipyrrin Complexes

**DOI:** 10.1021/acs.inorgchem.2c03048

**Published:** 2022-12-06

**Authors:** Karlotta van Rees, Thayalan Rajeshkumar, Laurent Maron, Stephen Sproules, Jason B. Love

**Affiliations:** † EaStCHEM School of Chemistry, The University of Edinburgh, Edinburgh EH9 3FJ, U.K.; ‡ LPCNO, INSA, Université de Toulouse, 135, Avenue de Rangueil, Toulouse Cedex 4 31077, France; § WestCHEM School of Chemistry, University of Glasgow, Glasgow G12 8QQ, U.K.

## Abstract

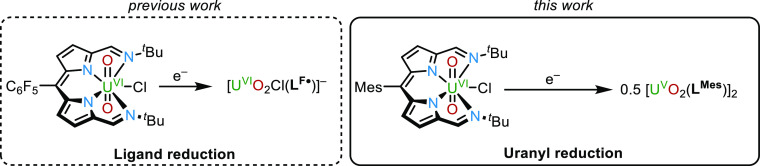

The uranyl complex U^VI^O_2_Cl(**L**^**Mes**^) of the redox-active, acyclic dipyrrin–diimine anion **L**^**Mes**–^ [H**L**^**Mes**^ = 1,9-di-*tert*-butyl-imine-5-(mesityl)dipyrrin] is reported, and its redox property is explored and compared with that of the previously reported U^VI^O_2_Cl(**L**^**F**^) [H**L**^**F**^ = 1,9-di-*tert*-butyl-imine-5-(pentafluorophenyl)dipyrrin] to understand the influence of the meso substituent. Cyclic voltammetry, electron paramagnetic resonance spectroscopy, and density functional theory studies show that the alteration from an electron-withdrawing meso substituent to an electron-donating meso substituent on the dipyrrin ligand significantly modifies the stability of the products formed after reduction. For U^VI^O_2_Cl(**L**^**Mes**^), the formation of a diamond-shaped, oxo-bridged uranyl(V) dimer, [U^V^O_2_(**L**^**Mes**^)]_2_ is seen, whereas in contrast, for U^VI^O_2_Cl(**L**^**F**^), only ligand reduction occurs. Computational modeling of these reactions shows that while ligand reduction followed by chloride dissociation occurs in both cases, ligand-to-metal electron transfer is favorable for U^VI^O_2_Cl(**L**^**Mes**^) only, which subsequently facilitates uranyl(V) dimerization.

## Abstract 2

Modifying the meso substituent on an acyclic uranyl(VI) dipyrrin−diimine complex from electron-withdrawing to electron-donating promotes uranium reduction instead of the expected ligand reduction. This modification causes the initially formed ligand radical to be unstable, with the complex undergoing ligand-to-metal electron transfer to form a uranyl(V) dimer.

## Introduction

The single-electron reduction of the ubiquitous and inert uranyl(VI) dication, UO_2_^2+^, is an important facet in environmental uranium remediation due to the easy disproportionation of the uranyl(V) cation, UO_2_^+^, into immobile uranium(IV).^1^ Significant advances have been made in the study of the direct reduction chemistry of uranyl(VI) using anaerobic techniques, resulting in a wide variety of isolable, often oxo-functionalized uranyl(V) complexes, some of which show significant stability in air.^2^

An alternative route to reduced uranium chemistry is to pair the uranyl(VI) cation with a redox-active ligand. Studies of uranyl(VI) complexes of redox-active ligands have been reported for Schiff bases,^3−5^ quinones,^6^ pyrroles, tetraaza[14]-annulenes,^7^ NacNac,^8^ calix[4]pyrroles,^9^ and dipyrrins.^10−12^ Recently, it was shown that uranyl(VI) complexes of pentadentate N_3_O_2_–saldien ligands underwent metal-based, one-electron reduction only, with a clear increase in the U^VI/V^ reduction potential associated with an increase in the electron-withdrawing nature of the substituents.^13^ In contrast, uranyl(VI) complexes of α-di-iminediphenolate or salophen ligands undergo single-electron ligand reductions, leading to the uranyl(VI) ligand-centered radical anions and not the expected uranyl(V) complexes.^3,4,14^ Lastly, uniquely redox-active and water stable uranyl(V) complexes of dipicolinate and aminocarboxylate ligands have been reported.^15^

**Scheme 1:**
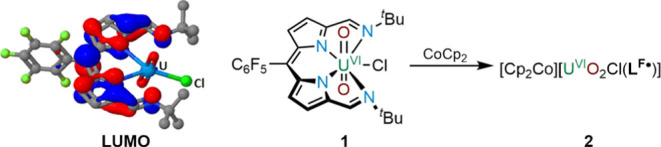
Previous Work Carried out on U^VI^O_2_Cl(**L**^**F**^), (**1**) The molecular orbital plot of **1**. The ISO value is 0.02 au. Positive is blue; negative is red.

We recently reported the redox behavior of uranyl(VI) complexes of the donor-expanded Schiff-base dipyrrin (**1**) (Scheme 1).^11,12^ The reaction of **1** with the outer-sphere reductant CoCp_2_ resulted in a single-electron reduction of the ligand to form the uranyl(VI) dipyrrin radical complex, [Cp_2_Co][U^VI^O_2_Cl(**L**^**F•**^)] (**2**); the addition of a second equivalent of CoCp_2_ reduced the uranium center to uranyl(V). In this case, the lowest unoccupied molecular orbital (LUMO) of **1** was found to be ligand-based, and while this favored outer-sphere ligand reduction, the metal reduction could be promoted using the inner-sphere reductant [Cp_2_TiCl]_2_ through Lewis acid activation of the uranyl oxo group, which diminished the U^VI/V^ reduction potential.

It is known that modifying the *meso*-carbon substituent of the dipyrrin ligand can influence geometry and chemistry due to steric and electronic effects.^16^ It was therefore envisaged that modifying the *meso*-carbon substituent in **1** from the electron-withdrawing C_6_F_5_ group to the electron-donating mesityl (C_6_H_2_Me_3_-2,4,6) may flip the redox chemistry of its uranyl complex, from ligand-based to metal-based. This study presents the formation of new uranyl(VI) complexes of a dipyrrin–diimine ligand and an evaluation of its reduction properties. The incorporation of the electron-donating mesityl meso substituent is found to change significantly the stability of the products formed after one-electron reduction.

## Experimental Section

### General Procedure

**Caution**: Depleted uranium (primary isotope ^238^U) is a weak α-emitter (4.197 MeV) with a half-life of 4.47 × 10^9^ years. Manipulations and reactions should be carried out in monitored fume hoods or in an inert atmosphere glovebox in a radiation laboratory equipped with α- and β-counting equipment.

The syntheses of all air- and moisture-sensitive compounds were carried out using standard Schlenk techniques under an atmosphere of dry argon. Vacuum atmospheres and MBraun gloveboxes were used to manipulate and store air- and moisture-sensitive compounds under an atmosphere of dried and deoxygenated dinitrogen. The solvents benzene-*d*_6_ and pyridine-*d*_5_ were refluxed over potassium metal overnight, trap-to-trap distilled, and free-pump–thaw degassed three times prior to use. All glassware was dried in an oven at 160 °C, cooled under 10^–3^ mbar vacuum, and then purged with argon. Prior to use, all Fisherbrand *R* 1.2 mm retention glass microfiber filters and stainless-steel cannulae were dried in an oven at 160 °C overnight. All solvents for use with air- and moisture-sensitive compounds were stored in Teflon-tapped ampoules containing pre-dried 4 Å molecular sieves. Dry solvents were collected from a solvent purification system (Innovation Technologies). All chemicals were used as received without any purification, unless otherwise specified. Tetrabutylammonium hexafluorophosphate, [^*n*^Bu_4_N][PF_6_], was recrystallized twice from absolute ethanol and dried for 2 days under vacuum.

^1^H NMR spectra were recorded on a Bruker AVA400 spectrometer operating at 399.90 MHz, a Bruker AVA500 or a Bruker PRO500 operating at 500.12 MHz, or a Bruker AVA600 spectrometer operating at 599.81 MHz. ^13^C{^1^H} NMR spectra were recorded on a Bruker AVA500 or a Bruker PRO500 operating at 125.76 MHz. ^19^F{^1^H} NMR spectra were recorded on a Bruker AVA500 spectrometer operating at 470.59 MHz. Chemical shifts are reported in parts per million (ppm). ^1^H and ^13^C{^1^H} NMR spectra are referenced to residual solvent resonances calibrated against the external standard, SiMe_4_ (δ = 0 ppm). ^19^F{^1^H} NMR spectra are referenced to the external standard, CCl_3_F (δ = 0 ppm). All spectra were recorded at 298 K unless otherwise specified. All data were processed using MestReNova 12.0.3. Full assignment of the NMR data is provided in the Supporting Information.

Single-crystal X-ray diffraction data were collected at 120 K on an Oxford Diffraction Excalibur diffractometer using graphite monochromated Mo K_α_ radiation equipped with an Eos charge-coupled device detector (λ = 0.71073 Å), or at 120 K on a Supernova, Dual, Cu at zero Atlas diffractometer using Cu K_α_ radiation (λ = 1.5418 Å). Structures were solved using ShelXT direct methods or intrinsic phasing and refined using a full-matrix least-squares refinement on |*F*|^2^ using ShelXL.^17^ All programs were used within the Olex suite.^18^ All non-hydrogen atoms were refined with anisotropic displacement parameters, and hydrogen atom parameters were constrained to parent atoms and refined using a riding model unless otherwise specified. All X-ray crystal structures were analyzed and illustrated using Mercury 4.3.1.

Elemental analyses were recorded in duplicate by Mr. Stephen Boyer at the London Metropolitan University and by Elemental Microanalysis Ltd. All Fourier transform infrared (FTIR) spectra were recorded using JASCO 410 or JASCO 460 plus spectrometers. Intensities are assigned as w = weak, m = medium, and s = strong. All UV–vis absorption spectra were recorded on a Jasco V-670 spectrometer on a 10 mm quartz cuvette, fitted with a septum for air-sensitive compounds.

### Synthesis

#### H**L**^**Mes**^

1,9-Diformyl-5-(mesityl)dipyrromethane^19^ (2.2 g, 6.9 mmol, 1 equiv) was dissolved in PhCH_3_ (300 mL). After the addition of Na_2_SO_4_ (4.4 g, 30.9 mmol, 4.5 equiv) and *tert*-butylamine (4.9 mL, 46.7 mmol, 6.8 equiv), the reaction mixture was heated at 50 °C for 48 h. The mixture was filtered, and the solvent was removed under reduced pressure, leaving behind a dark red oil, which was redissolved *n*-hexane (25 mL). The formed black solid was removed *via* filtration, and the remaining solvent was removed under reduced pressure, yielding H**L**^**Mes**^ as a dark red solid. Yield = 2.4 g (82%). Reddish-brown block-shaped crystals of H**L**^**Mes**^ suitable for single-crystal X-ray diffraction were grown at −20 °C from a concentrated CH_2_Cl_2_ solution. ^1^H NMR (500 MHz, chloroform-*d*): δ_H_ 12.63 (br s, 1H, N*H*), 8.30 (s, 2H, imine), 6.92 (s, 2H, *m*-Mes-C*H*), 6.71 (d, *J* = 4.3 Hz, 2H, β-pyrrole), 6.38 (d, *J* = 4.2 Hz, 2H, β-pyrrole), 2.35 (s, 3H, *p*-Mes-CC*H*_*3*_), 2.08 (s, 6H, *o*-Mes-CC*H*_*3*_), 1.32 (s, 18H, ^*t*^Bu-C(C*H*_*3*_)_3_). ^13^C{^1^H} NMR (126 MHz, chloroform-*d*): δ_C_ 153.65 (α-pyrrole), 149.38 (imine), 142.57 (α-pyrrole), 140.43 (*o*-Mes-*C*CH_3_), 137.66 (*p*-Mes-*C*CH_3_), 136.70 (*ipso*-Mes), 133.10 (*meso*-*C*), 128.10 (*m*-Mes-*C*H), 127.87 (β-pyrrole), 118.74 (β-pyrrole), 57.82 (^*t*^Bu-*C*(CH_3_)_3_), 29.67 (^*t*^Bu-C(*C*H_3_)_3_), 21.12 (*p*-Mes-C*C*H_3_), 19.93 (*o*-Mes-C*C*H_3_). FTIR (film) ν_max_: 1576 cm^–1^. UV–vis (THF): λ_max_ 272.5 nm, ε = 55 206 M^–1^ cm^–1^; λ 476 nm, ε = 36 961 M^–1^ cm^–1^. Elemental analysis: C_28_H_36_N_4_ (MW = 428.3 g mol^–1^) requires C, 78.46; H, 8.47; N, 13.07%. Found: C, 78.22; H, 8.61; N, 12.91%. MS (MALDI-TOF, ACN) *m*/*z*: [MH]^+^ requires 429.301, found 429.301. HRMS (ESI^+^, EtOH) *m*/*z*: C_28_H_37_N_4_ [M + H]^+^ requires 429.30127, found 429.30120 (mass error = −0.07 ppm).

#### K(**L**^**Mes**^)

The synthesis was conducted under an inert atmosphere. In an ampoule, KH (16 mg, 0.4 mmol, 1.5 equiv) was suspended in anhydrous tetrahydrofuran (THF) (10 mL) and cooled to 0 °C. A solution of H**L**^**Mes**^ in THF (110 mg, 0.3 mmol, 1 equiv; 10 mL) was added dropwise, and the mixture was allowed to slowly warm to room temperature (RT), causing the reaction mixture to slowly change color from dark orange brown to pinkish purple. The solution was stirred for 16 h at RT before being filtered. The solvent was evaporated under reduced pressure, leaving a golden purple solid that was subsequently dried overnight under reduced pressure at 55 °C. Yield = 100 mg (86%). Greenish-pink needle-shaped crystals suitable for single-crystal X-ray diffraction were obtained at −20 °C from an *n*-hexane/THF solution (1:1). ^1^H NMR (500 MHz, benzene-*d*_6_): δ_H_ 8.18 (m, 2H, imine), 6.99 (s, 2H, *m*-Mes-C*H*), 6.92 (m, 2H, β-pyrrole), 6.76 (m, 2H, β-pyrrole), 2.46 (s, 6H, *o*-Mes-CC*H*_*3*_), 2.29 (s, 3H, *p*-Mes-CC*H*_*3*_), 0.99 (s, 18H, ^*t*^Bu-C(C*H*_*3*_)_3_). ^13^C{^1^H} NMR (126 MHz, benzene-*d*_6_): δ_C_ 156.24 (α-pyrrole), 154.01 (imine), 152.64 (α-pyrrole), 145.97 (*o*-Mes-*C*CH_3_), 139.20 (*p*-Mes-*C*CH_3_), 136.55 (*ipso*-Mes), 136.04 (*meso*-*C*), 130.64 (β-pyrrole), 127.98 (*m*-Mes-*C*H), 120.91 (β-pyrrole), 55.91 (^*t*^Bu-C(CH_3_)_3_), 29.70 (^*t*^Bu-C(*C*H_3_)_3_), 20.92 (*p*-Mes-C*C*H_3_), 20.07 (*o*-Mes-C*C*H_3_). UV–vis (THF): λ_max_ 568 nm, ε = 46 315 M^–1^ cm^–1^; λ 483 nm, ε = 15 146 M^–1^ cm^–1^; λ 297 nm, ε = 22 163 M^–1^ cm^–1^; λ 275 nm, ε = 24 385 M^–1^ cm^–1^; λ 222 nm, ε = 18 654 M^–1^ cm^–1^. Elemental analysis: C_28_H_35_KN_4_ (MW = 466.3 g mol^–1^) requires C, 72.06; H, 7.56; N, 12.00%. Found: C, 66.34; H, 7.27; N, 10.45% (unsatisfactory due to the rapid hydrolysis of the complex). HRMS (APPI^+^, THF) *m*/*z*: C_28_H_36_KN_4_ [M + H]^+^ requires 467.25716, found 467.257770 (mass error = 1.30 ppm).

#### U^VI^O_2_Cl(**L**^**Mes**^)

**Method A**: K(**L**^**Mes**^) was prepared *in situ* by the synthesis process described above using KH (71 mg, 1.8 mmol, 1.5 equiv) and H**L**^**Mes**^ in anhydrous THF (490 mg, 1.2 mmol, 1 equiv; 10 mL). The solution was stirred for 16 h before being filtered into a Schlenk tube containing a solution of U^VI^O_2_Cl_2_(THF)_2_ in THF (560 mg, 1.2 mmol, 1 equiv; 5 mL) and stirred for an additional 16 h, during which the mixture turned deep purple. The mixture was filtered, and the solvent was evaporated under reduced pressure, leaving U^VI^O_2_Cl(**L**^**Mes**^) as a deep purple sold. Yield = 810 mg (94%). Golden-pink block-shaped crystals suitable for single-crystal X-ray diffraction were grown at −20 °C from an *n*-hexane/THF solution (1:1). ^1^H NMR (500 MHz, benzene-*d*_6_): δ_H_ 8.80 (s, 2H, imine), 6.90 (d, *J* = 4.2 Hz, 2H, β-pyrrole), 6.86–6.81 (m, 2H, *m*-Mes-C*H*), 6.59 (d, *J* = 4.2 Hz, 2H, β-pyrrole), 2.24 (s, 3H, *p*-Mes-CC*H*_*3*_), 2.15 (s, 6H, *o*-Mes-CC*H*_*3*_), 1.92 (s, 18H, ^*t*^Bu-C(C*H*_*3*_)_3_). ^13^C{^1^H} NMR (126 MHz, benzene-*d*_6_): δ_C_ 157.79 (α-pyrrole), 157.54 (imine), 153.86 (*meso*-*C*), 147.26 (α-pyrrole), 137.82 (*p*-Mes-*C*CH_3_), 136.57 (*ipso*-Mes), 135.06 (*o*-Mes-*C*CH_3_), 133.75 (β-pyrrole), 127.98 (*m*-Mes-*C*H), 122.79 (β-pyrrole), 64.87 (tBu-*C*(CH_3_)_3_), 30.37 (^*t*^Bu-C(*C*H_3_)_3_), 20.81 (*p*-Mes-C*C*H_3_), 19.70 (*o*-Mes-C*C*H_3_). UV–vis (THF): λ_max_ 584.5 nm, ε = 9210 M^–1^ cm^–1^; λ 539 nm, ε = 5526 M^–1^ cm^–1^; λ 292 nm, ε = 15 421 M^–1^ cm^–1^. Elemental analysis: C_28_H_35_ClN_4_O_2_U (MW = 732.3 g mol^–1^) requires C, 45.88; H, 4.81; N, 7.64%. Found: C, 45.55; H, 4.91; N, 6.94%. HRMS (APPI^+^, THF) *m*/*z*: C_28_H_36_UO_2_N_4_Cl [M + H]^+^ requires 733.30291, found 733.307575 (mass error = 6.36 ppm); C_28_H_35_UO_2_N_4_ [M – Cl]^+^ requires 697.32624, found 697.326611 (mass error = 0.53 ppm).

#### [U^V^O_2_(**L**^**Mes**^)]_2_

The synthesis was conducted under an inert atmosphere. A deep purple solution of U^VI^O_2_Cl(**L**^**Mes**^) in C_6_D_6_ (100 mg, 0.1 mmol, 1 equiv; 2 mL) was added to a solution of CoCp_2_ in benzene (25 mg, 0.1 mmol, 1 equiv; 2 mL). The solution was stirred for 1 h at RT, during which a golden purple precipitate formed, which was isolated by centrifuging. Yield = 68 mg (76%). Golden-pink plate-shaped crystals suitable for single-crystal X-ray diffraction of [U^V^O_2_(**L**^**Mes**^)]_2_ were grown by slowly cooling a heated concentrated benzene-*d*_6_ solution in a Teflon-tapped NMR tube. ^1^H NMR (500 MHz, pyridine-*d*_5_): δ_H_ 3.14 (s, 1H, *m*-Mes-C*H*), 1.63 (s, 1H, *m*-Mes-C*H*), −0.33 (br s, 3H, *p*-Mes-CC*H*_*3*_), −0.59 (s, 3H, *o*-Mes-CC*H*_*3*_), −1.90 (s, 3H, *o*-Mes-CC*H*_*3*_), −4.94 (br s, 2H, β-pyrrole), −5.41 (br s, 2H, β-pyrrole), −6.12 (br s, 12H, ^*t*^Bu-C(C*H*_*3*_)_3_), −6.22 (br s, 6H, ^*t*^Bu-C(C*H*_*3*_)_3_), −9.17 (s, 2H, imine). ^13^C{^1^H} NMR (126 MHz, pyridine-*d*_5_): δ_C_ 132.19, 126.82, 122.15, 121.28, 118.63, 116.91, 101.73, 77.22, 76.00, 67.60, 32.52, 29.39, 25.58, 20.11, 17.50, 16.04, 14.48. HRMS (APPI^+^, THF) *m*/*z*: C_56_H_70_N_8_O_4_U_2_ [M]^+^ requires 1394.653033, found 1394.668864 (mass error = 11.35 ppm); C_28_H_35_UO_2_N_4_ [0.5M]^+^ requires 697.32624, found 697.327537 (mass error = 1.85 ppm). Elemental analysis: C_56_H_70_N_8_O_4_U_2_ (MW = 1395.29 g mol^–1^) requires C, 48.21; H, 5.06; N, 8.03%. Found: C, 48.55; H, 5.27; N, 8.19%.

## Results

### Synthesis and Structure of Uranyl(VI) Complexes

**Scheme 2:**
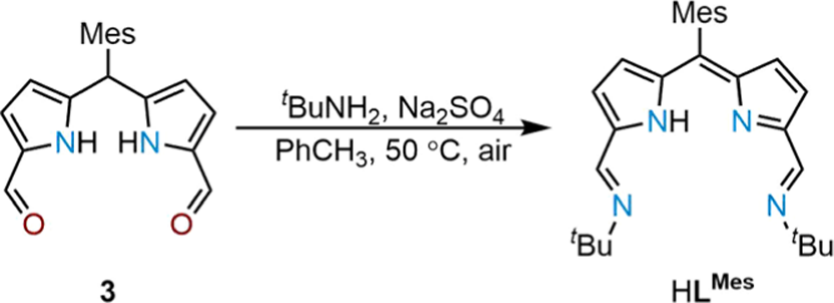
Synthesis of Ligand H**L**^**Mes**^

The dipyrrin ligand H**L**^**Mes**^ is obtained in 82% yield through a straightforward aerobic condensation/oxidation reaction between the mono-meso-substituted dipyrromethane dialdehyde **3** and excess *tert*-butylamine in toluene at RT (Scheme 2). The ^1^H NMR spectrum of H**L**^**Mes**^ depicts an imine proton resonance at 8.30 ppm and two resonances at 2.35 and 2.08 ppm for the mesityl group, indicating a *C*_2*v*_ symmetry in solution. Two doublets at 6.72 and 6.39 ppm are assigned to the β-pyrrole protons, and the singlet at 1.32 ppm is assigned to the *tert*-butyl group. In addition, the disappearance of the *meso*-proton resonance reveals that spontaneous oxidation of the dipyrromethane to the dipyrrin has occurred, similar to that seen previously in the synthesis of other Schiff base dipyrrins.^20^

**Figure 1:**
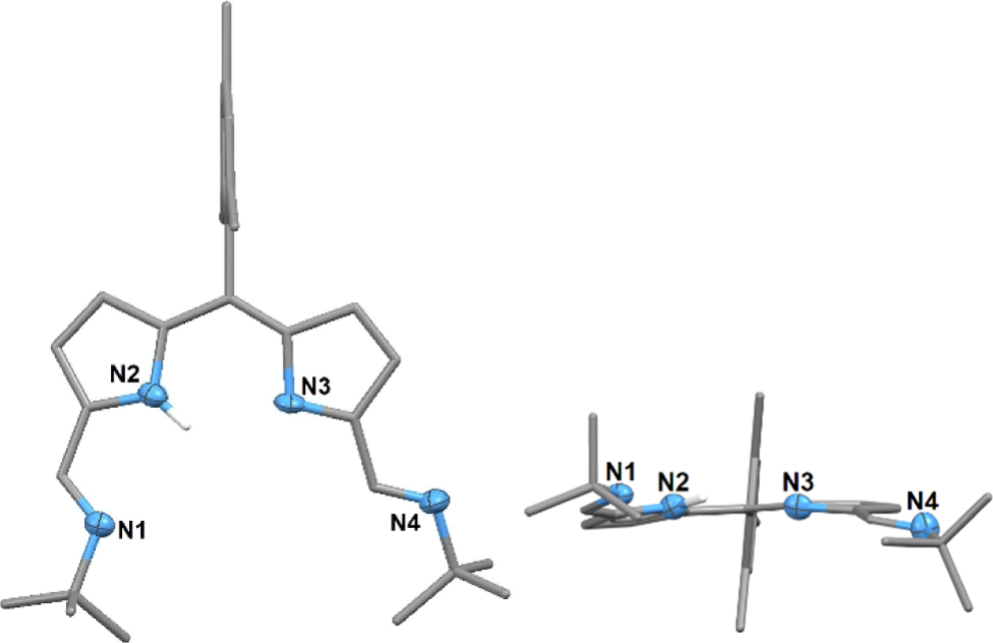
Solid-state structure of H**L**^**Mes**^. For clarity, all hydrogen atoms except that of NH are omitted (where shown, displacement ellipsoids are drawn at 50% probability). Carbon atoms are gray.

Reddish-brown block-shaped single crystals of H**L**^**Mes**^ suitable for X-ray diffraction were grown from a concentrated diethyl ether solution at −30 °C (Figure 1). While the data are poor, the connectivity is clear with the planar sp^2^ hybridized *meso*-carbon further confirming the spontaneous oxidation of the ligand during its synthesis.

The reaction between H**L**^**Mes**^ and 1 equiv of KH in anhydrous THF cleanly generates the potassium complex K(**L**^**Mes**^), which is isolated as a golden purple solid in 86% yield. The ^1^H NMR spectrum of K(**L**^**Mes**^) shows the disappearance of the NH proton, while the imine proton resonance is at 8.18 ppm and the β-pyrrole protons appear at 6.92 and 6.76 ppm. The mesityl methyl protons appear at 2.46 and 2.29 ppm, indicative of top/bottom symmetry.

**Figure 2:**
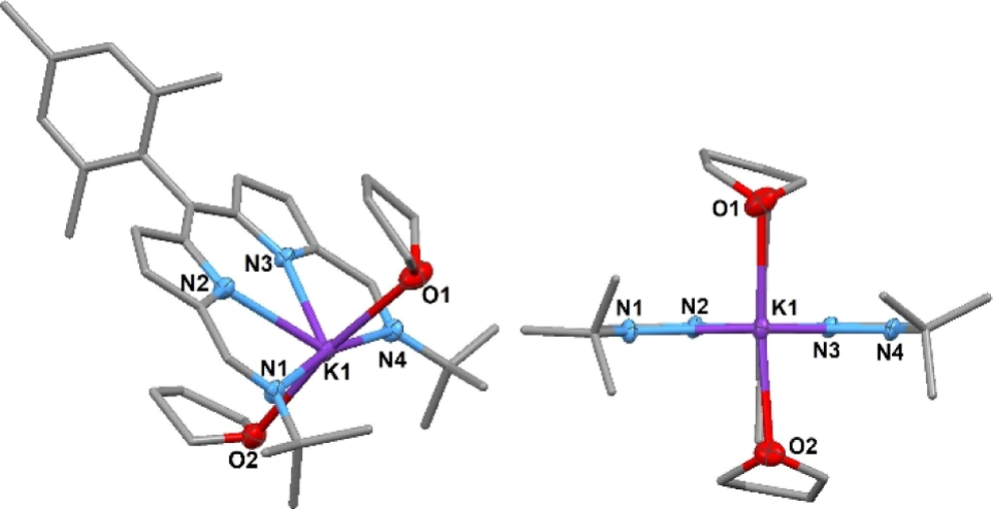
Solid-state structure of K(**L**^**Mes**^)·(THF)_2_ viewed from the top (left) and side (right). For clarity, all hydrogen atoms and one molecule are omitted (displacement ellipsoids are drawn at 50% probability). Selected bonds (Å) and angles (deg): K1–N1, 2.897(3); K1–N2, 2.732(4); K1–N3, 2.715(3); K1–N4, 2.916(3); K1–O1, 2.761(3); K1–O2, 2.799(3); N1–K1–N2, 61.65(9); N2–K1–N3, 66.40(9); N3–K1–N4, 62.28(9); N4–K1–N1, 169.56(9); O1–K1–O2, 171.36(9); C20–C10–C9, 128.2(4); C9–C10–C11, 116.5(4); and C11–C10–C20, 115.3(4).

Greenish-purple needle-shaped crystals of K(**L**^**Mes**^) suitable for X-ray diffraction were grown from a concentrated 1:1 THF/*n*-hexane solution at −30 °C (Figure 2). The crystal is the THF solvate of K(**L**^**Mes**^) and exhibits a distorted octahedral geometry with the ligand coordinating in the equatorial plane in an N_4_ coordination mode. There is no steric hindrance between the ligand and the coordinated potassium metal, indicated by the insignificant distance of 0.035 Å between the plane of the N_4_ donor set and the potassium atom.

**Scheme 3:**
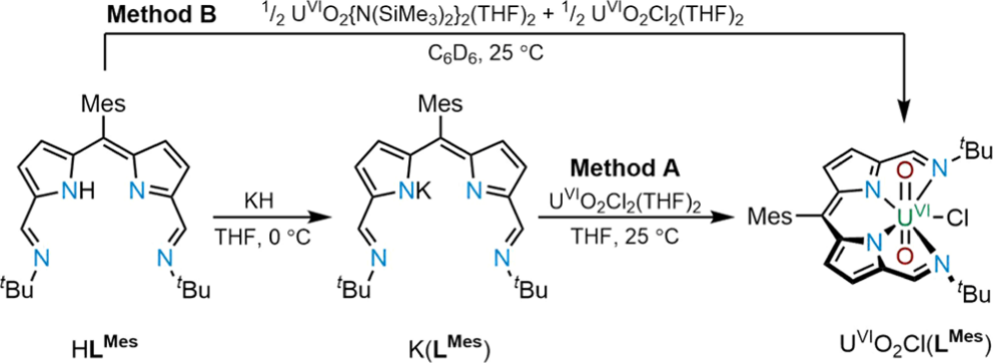
Synthesis of U^VI^O_2_Cl(**L**^**Mes**^) by Transmetalation with K(**L**^**Mes**^) (Method A) and Directly from H**L**^**Mes**^ (Method B)

The uranyl complex U^VI^O_2_Cl(**L**^**Mes**^) was prepared by two different methods. Method A is a transmetalation reaction between 1 equiv of K(**L**^**Mes**^) with an equimolar amount of U^VI^O_2_Cl_2_(THF)_2_ (Scheme 3), whereas method B reacts H**L**^**Mes**^ with a 1:1 mixture of U^VI^O_2_{N(SiMe_3_)_2_}_2_(THF)_2_ and U^VI^O_2_Cl_2_(THF)_2_ in benzene (see the Supporting Information). The ^1^H NMR spectrum of U^VI^O_2_Cl(**L**^**Mes**^) has an imine resonance at 8.80 and the β-pyrrole protons at 6.90 and 6.59 ppm. Two singlets corresponding to the mesityl methyl groups at 2.24 and 2.15 ppm are indicative of *C*_2*v*_ symmetry.

**Figure 3:**
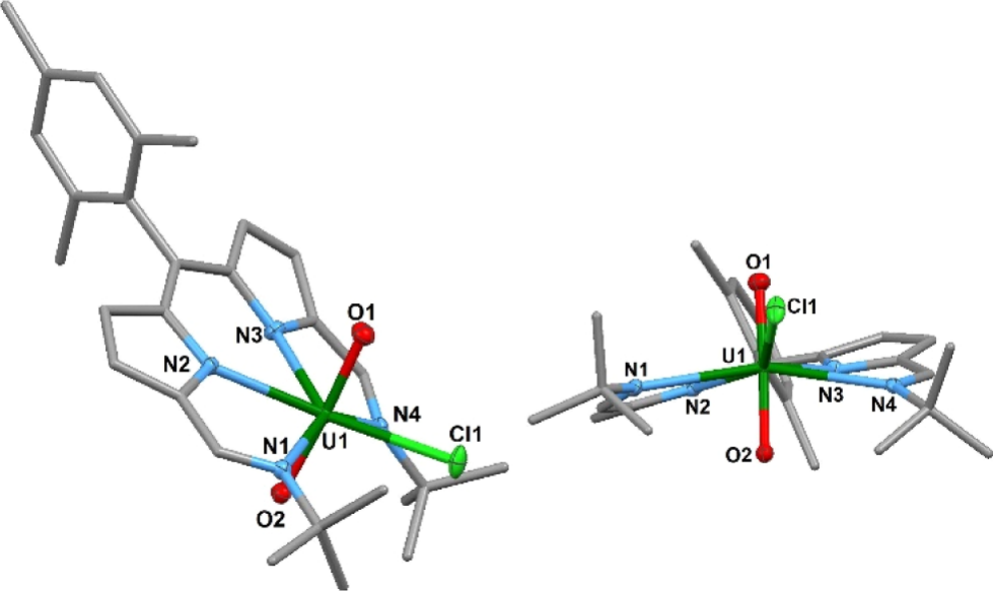
Solid-state structure of U^VI^O_2_Cl(**L**^**Mes**^) viewed from the top (left) and side (right). For clarity, all hydrogen atoms are omitted (displacement ellipsoids are drawn at 50% probability). Selected bonds distances (Å) and angles (deg): U1–N1, 2.676(2); U1–N2, 2.469(2); U1–N3, 2.477(2); U1–N4, 2.675(2); U1–O1, 1.765(2); U1–O2, 1.768(2); U1–Cl1, 2.6882(7); N1–U1–N2, 65.85(6); N2–U1–N3, 70.30(6); N3–U1–N4, 65.30(6); N4–U1–N1, 152.03(6); N4–U1–Cl1, 79.22(4); N1–U1–Cl1, 87.84(4); and O1–U1–O2, 176.15(8).

Purplish-golden block-shaped single crystals of U^VI^O_2_Cl(**L**^**Mes**^) were grown from a concentrated 1:1 THF/*n*-hexane solution at −30 °C (Figure 3). In the solid state, the uranium center adopts a distorted pentagonal bipyramidal coordination geometry in which the N_4_ donor set of the expanded dipyrrin ligand occupies the equatorial positions along with the chloride ligand; this structure is similar to that of U^VI^O_2_Cl(**L**^**F**^).^11^ The Cl1 atom is situated 1.621 Å above the mean N_4_ plane and indicates a steric interaction between this ligand and the nearby *tert*-butyl groups. These *tert*-butyl groups bend away from the same face of the N_4_ donor plane, meaning that the *C*_2*v*_ symmetry observed in the solution state is not retained in the solid state; this feature was also seen in U^VI^O_2_Cl(**L**^**F**^).^11^ The uranium oxo bond distances O1–U1 and U1–O2 are 1.765(2) and 1.768(2) Å, respectively, with an O1–U1–O2 angle of 176.15(8)°. This complex exhibits U=O bond lengths and O=U=O angles in the range of other non-functionalized uranyl(VI) complexes reported since 2010,^2^ in which the average U=O bond length is 1.777 Å. The U1–N_pyrrole_ bond lengths are 2.469(2) and 2.477(2) Å, while the U1–N_imine_ bond lengths are 2.676(2) and 2.675(2) Å. The U1–Cl1 bond length is 2.6882(7) Å and similar to that seen in U^VI^O_2_Cl(**L**^**F**^).^11^

### Electronic Spectroscopy

**Figure 4:**
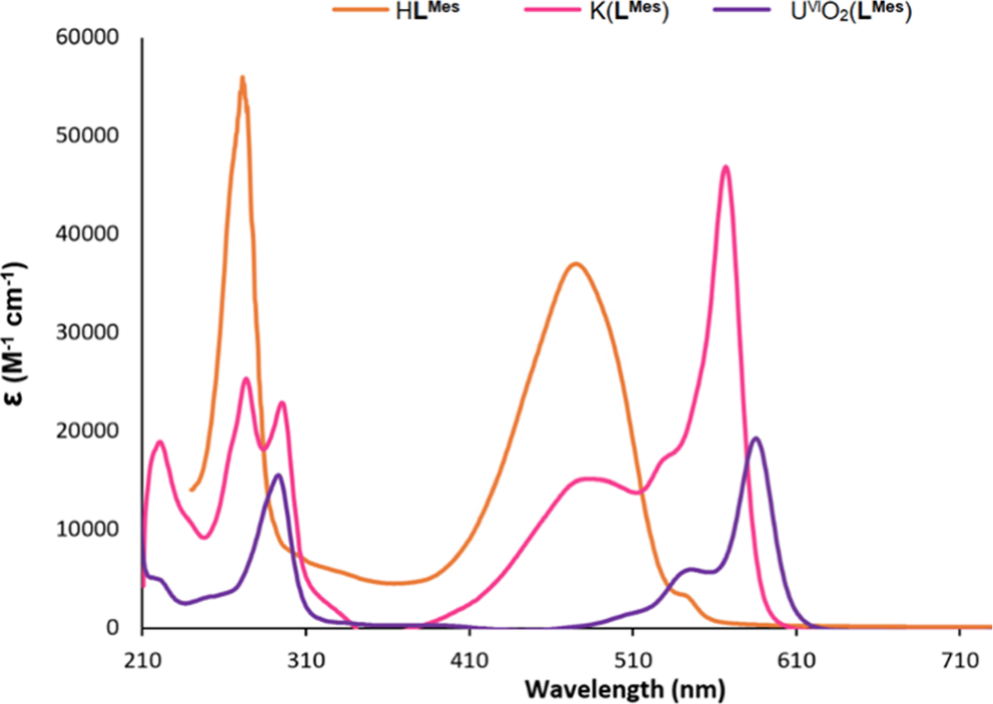
UV–vis spectra of H**L**^**Mes**^, K(**L**^**Mes**^), and U^VI^O_2_Cl(**L**^**Mes**^) in anhydrous THF.

The absorbance spectra of H**L**^**Mes**^, K(**L**^**Mes**^), and U^VI^O_2_Cl(**L**^**Mes**^) were recorded in anhydrous THF (Figure 4). H**L**^**Mes**^ has a maximum absorbance of 272 nm (ε = 55 206 M^–1^ cm^–1^) and a second peak at 476 nm (ε = 36 961 M^–1^ cm^–1^), which are similar to that for H**L**^**F**^. Although no time-dependent density functional theory (TD-DFT) calculations have been conducted, the latter absorption band is likely attributed to the ligand-centered π → π* transition localized on the dipyrrin–diimine fragment. Upon metalation to form the potassium salt K(**L**^**Mes**^), the easy to visualize color change is reflected in the UV–vis spectrum with significant red shifts observed relative to that of H**L**^**Mes**^ with a maximum absorbance at 568 nm (ε = 46 315 M^–1^ cm^–1^). The uranyl complex U^VI^O_2_Cl(**L**^**Mes**^) is red-shifted further and with a decrease in the extinction coefficient, exhibiting a maximum absorbance at 584 nm (ε = 19 210 M^–1^ cm^–1^) with a shoulder at 539 nm (ε = 5526 M^–1^ cm^–1^).

### Synthesis and Structure of the Uranyl(V) Dimer

The reaction between U^VI^O_2_Cl(**L**^**Mes**^) and 1 equiv of CoCp_2_ in benzene results in the precipitation of a golden pink paramagnetic species, which is soluble in pyridine (Scheme 3). The ^1^H NMR spectrum in pyridine-*d*_5_ exhibits resonances between +4 and −10 ppm, consistent with the reduction of uranyl(VI) to uranyl(V) and not the formation of a ligand radical. The spectrum depicts five individual mesityl peaks as a result of top/bottom asymmetry. The mesityl CH peaks shift to 3.14 and 1.96 ppm, and the methyl peaks shift to −0.33, −0.59, and −1.90 ppm. The β-pyrrole protons are seen at −4.94 and −5.41 ppm, and the imine proton is observed at −9.17 ppm. The *tert*-butyl protons are seen as two singlets at −6.12 and −6.21 ppm, having an area of 2:1, and is consistent with *C*_2_ symmetry.

**Figure 5:**
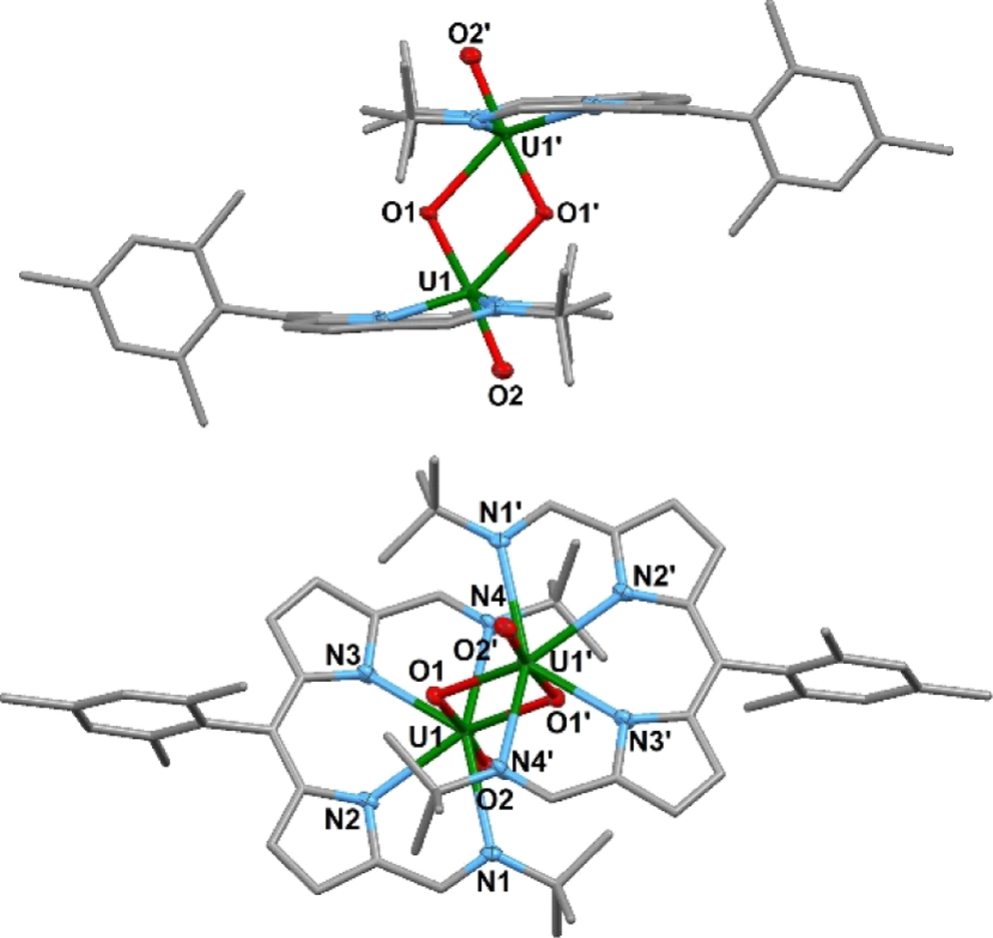
Solid-state structure of [U^V^O_2_(**L**^**Mes**^)]_2_ viewed from the side (top) and top (bottom). For clarity, one molecule, one benzene solvate molecule, and all hydrogen atoms are omitted (displacement ellipsoids are drawn at 50% probability). Carbon atoms are gray. Selected bond distances (Å) and angles (deg): U1–U1′, 3.5299(4); U1–N1, 2.694(3); U1–N2, 2.495(3); U1–N3, 2.502(3); U1–N4, 2.665(4); U1–O1, 1.933(3); U1–O2, 1.833(3); U1–O1′, 2.395(3); N1–U1–N2, 65.4(1); N2–U1–N3, 70.6(1); N3–U1–N4, 65.2(1); N4–U1–N1, 150.6(1); O1–U1–O2, 175.5(1); O1–U1–O1′, 70.7(1); O1′–U1–O2, 113.3(1); U1–O1′–U1′, 109.3(1); and U1–O1–U1′, 109.3(1).

Goldish-purple plate-shaped crystals were grown by slowly cooling a hot benzene solution, and the solid-state structure of [U^V^O_2_(**L**^**Mes**^)]_2_ was determined by X-ray crystallography. The solid-state structure reveals the formation of a uranyl(V) dimer complex [U^V^O_2_(**L**^**Mes**^)]_2_ and neither the formation of [Cp_2_Co][U^V^O_2_Cl(**L**^**Mes**^)] nor that of [Cp_2_Co][U^VI^O_2_Cl(**L**^**Mes•**^)] (Figure 5). The solid-state structure shows a diamond-shaped, dioxo-bridge between the two uranium(V) centers. The axial O1–U1 and O2–U1 bond lengths are 1.933(3) and 1.833(3) Å, respectively, and the equatorial O1′–U1 is longer at 2.395(3) Å. The O1–U1–O2 has a bond angle of 175.52°, O2–U1–O1′ has an angle of 113.31°, and O1′–U1–O1 has an angle of 70.74° with a U1···U1′ separation of 3.5389(4) Å. The U–O bond lengths of uranyl(V) dioxo complexes reported since 2010 range from 1.77(1) to 2.170(8) Å.^2^

A similar diamond-shaped, dioxo-bridged dimer has been synthesized before from U^VI^O_2_Cl(**L**^**F**^) through its reaction with 1 equiv of KNHDIPP (Dipp = 2,6-^*i*^Pr_2_C_6_H_3_).^12^ This reduction reaction presumably proceeded through the formation of the transient anilide complex U^VI^O_2_(NHDipp)(**L**^**F**^), which then underwent U–N bond homolysis.^12,21^ The solid-state structure of [U^V^O_2_(**L**^**F**^)]_2_ has similar metrics to [U^V^O_2_(**L**^**Mes**^)]_2_.^12^ The above-described dimers, or the coordination of an actinyl “yl” oxygen to the metal center of another actinyl fragments, are examples of cation–cation interactions, or CCIs, seen in actinide oxo complexes.^22^

### Electrochemistry

**Figure 6:**
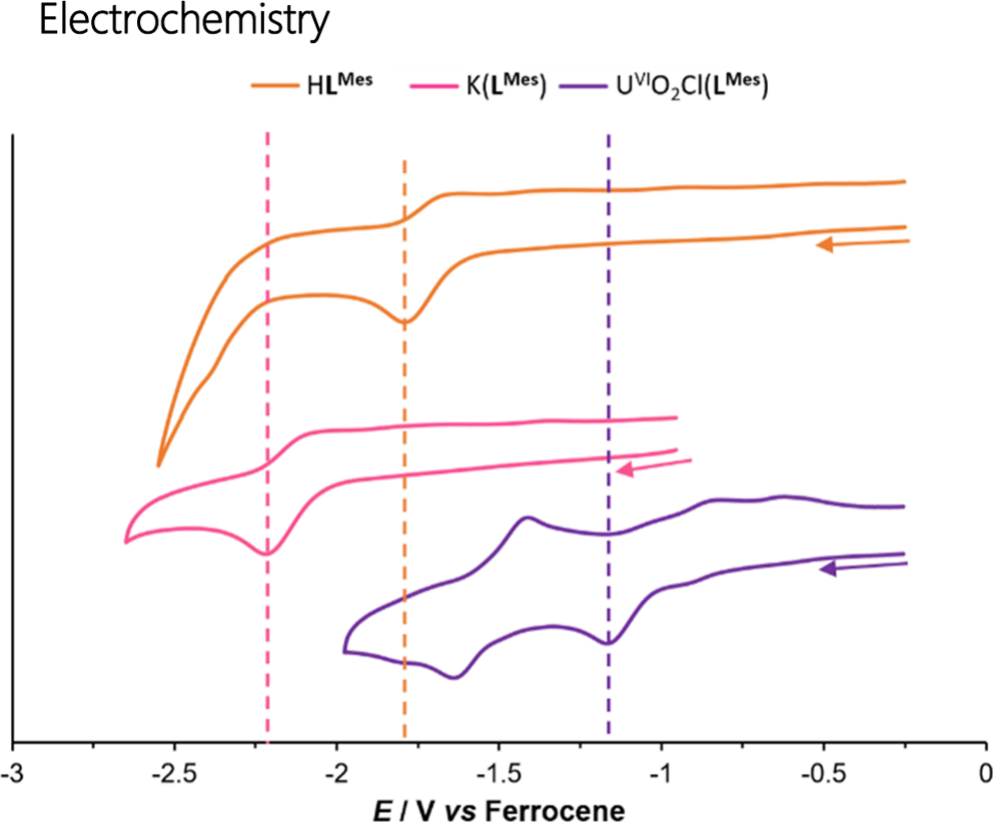
Stacked CVs of H**L**^**Mes**^, K(**L**^**Mes**^), and U^VI^O_2_Cl(**L**^**Mes**^). All were measured as 1 mM anhydrous CH_2_Cl_2_ solutions (a 1.0 M [^*n*^Bu_4_N][PF_6_] supporting electrolyte, a glassy carbon working electrode, a Pt gauze counter electrode, and a silver wire quasi-reference electrode). Potentials are referenced against Fc/Fc^+^ couple recorded under identical conditions.

The cyclic voltammograms (CVs) of H**L**^**Mes**^, K(**L**^**Mes**^) and U^VI^O_2_Cl(**L**^**Mes**^) were recorded in anhydrous CH_2_Cl_2_ at a scan rate of 100 mV s^–1^ (Figure 6). The CV of H**L**^**Mes**^ features a quasi-reversible reduction at *E*_1/2_ −1.72 V *versus* Fc/Fc^+^ and an irreversible reduction at *E*_pc_ −2.40 V *versus* Fc/Fc^+^. In comparison, H**L**^**F**^ displayed a significantly less-negative reduction of *E*_pc_ −1.51 V *versus* Fc/Fc^+^, showing that the electron-withdrawing *meso*-carbon substituent facilitates ligand reduction.^11^ The CV of K(**L**^**Mes**^) features a single quasi-reversible reduction at *E*_1/2_ −2.15 V *versus* Fc/Fc^+^.

The first reduction peaks of H**L**^**Mes**^ and K(**L**^**Mes**^) are quasi-reversible, and it can therefore be concluded that the radical species [H**L**^**Mes•**^]^−^ and [K(**L**^**Mes•**^)]^−^ are unstable under the cyclic voltammetry conditions. These features are more reversible with an increased scan rate (see the Supporting Information).

The CV of U^VI^O_2_Cl(**L**^**Mes**^) features two different redox processes upon cathodic scanning, the first being an irreversible wave at −1.15 V *versus* Fc/Fc^+^ and the second being a quasi-reversible reduction wave at −1.54 V *versus* Fc/Fc^+^. This is different from the CV of U^VI^O_2_Cl(**L**^**F**^) since this compound features three quasi-reversible reduction processes at *E*_1/2_ of–0.96, −1.18, and −2.02 V *versus* Fc/Fc^+^, corresponding with the ligand reduction, U^VI/V^, and U^V/IV^ reduction, respectively (Table 1). The CV of U^VI^O_2_Cl(**L**^**Mes**^) does, however, bear resemblance to that of the cationic compound [U^VI^O_2_(**L**^**F**^)][BAr^F^] (BAr^F^ = tetrakis[3,5-bis(trifluoromethyl)phenyl]borate),^12^ which showed an irreversible reduction at −0.64 V *versus* Fc/Fc^+^ and a quasi-reversible reduction wave at −1.24 V. The first reduction of [U^VI^O_2_(**L**^**F**^)][BAr^F^] was assigned as the U^VI^/U^V^ couple with its irreversibility indicating the formation of the U^V^ dimer [U^V^O_2_(**L**^**F**^)]_2_; the second peak was assigned as reduction to U^IV^.^12^

**Table 1:** Cyclic Voltammetry Data

complex	process	*E*_pc_ (V)	*E*_pa_ (V)	Δ*E* (V)	*E*_1/2_ (V)	reversibility	red/ox	assignment
H**L**^**Mes**^	I	–1.80	–1.66	0.14	–1.72	quasi-reversible	reduction	**L**^–^/**L**^**•**–^
	II	–2.40				irreversible	reduction	**L**^**•**–^/**L**^3–^
H**L**^**F**^^11^	I	–1.51				Irreversible	reduction	**L**^–^/**L**^**•**–^
	II	–2.02				irreversible	reduction	**L**^**•**–^/**L**^3–^
K(**L**^**Mes**^)	I				–2.15	quasi-reversible	reduction	**L**^–^/**L**^**•**–^
K(**L**^**F**^)^11^	I	–1.29				irreversible	reduction	**L**^–^/**L**^**•**–^
	II	–1.57				irreversible	reduction	**L**^**•**–^/**L**^3–^
U^VI^O_2_Cl(**L**^**Mes**^)	I	–1.15				irreversible	reduction	**L**^–^/**L**^**•**–^
	II	–1.64	–1.43	0.21	–1.54	quasi-reversible	reduction	U^VI^/U^V^
U^VI^O_2_Cl(**L**^**F**^)^11^	I	–1.03	–0.89	0.14	–0.96	quasi-reversible	reduction	**L**^–^/**L**^**•**–^
	II	–1.25	–1.10	0.15	–1.18	quasi-reversible	reduction	U^VI^/U^V^
	III	–2.10	–1.94	0.16	–2.02	quasi-reversible	reduction	U^V^/U^IV^
[U^VI^O_2_(**L**^**F**^)][BarF]^12^	I	–0.64				irreversible	reduction	U^VI^/U^V^
	II	–1.37	–1.12	0.25	–1.24	quasi-reversible	reduction	U^V^/U^IV^

### Electron Paramagnetic Resonance Spectroscopy

**Figure 7:**
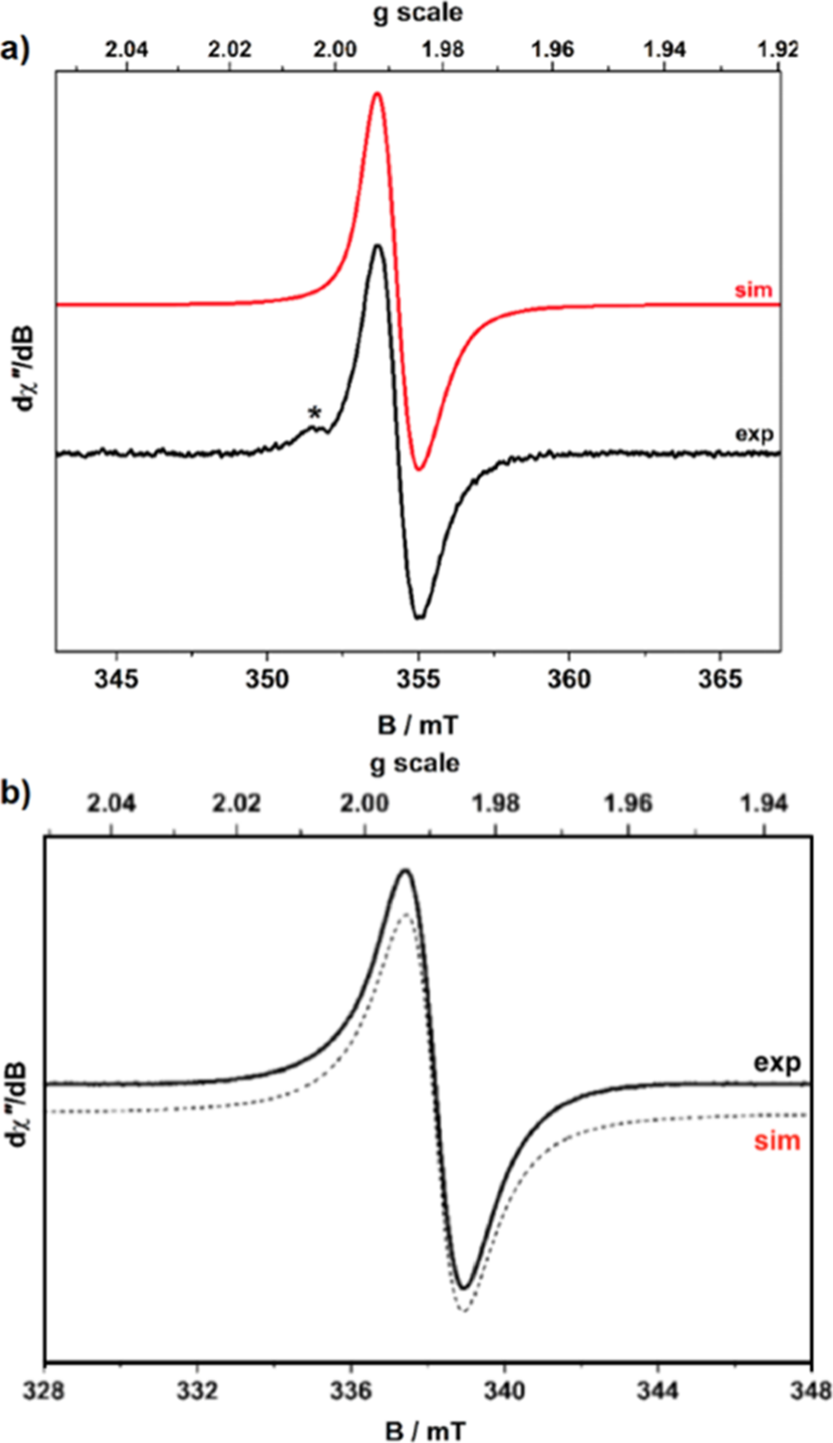
X-Band EPR spectra of [U^VI^O_2_Cl(**L**^**Mes•**^)]^−^ (a) and [U^VI^O_2_Cl(**L**^**F•**^)]^−^ (b) generated in anhydrous CH_2_Cl_2_ solution at ambient temperature. The measured spectra are shown in black solid lines, and the simulated spectra are shown in dashed black and solid red lines.

Although the CVs depicted similarities between U^VI^O_2_Cl(**L**^**Mes**^) and [U^VI^O_2_(**L**^**F**^)][BAr^F^], electron paramagnetic resonance (EPR) analysis was still carried out on the reduction of U^VI^O_2_Cl(**L**^**Mes**^) to rule out ligand reduction. As such, U^VI^O_2_Cl(**L**^**Mes**^) was reacted with CoCp_2_ in anhydrous CH_2_Cl_2_ at ambient temperature and monitored. The EPR shows the formation of [U^VI^O_2_Cl(**L**^**Mes•**^)]^−^ with a *g*_iso_ value of 1.987 (Figure 7). There is unresolved hyperfine coupling that gives rise to this unique line shape that is the consequence of perturbed molecular tumbling in solution and line broadening driven by spin–orbital contribution from U^VI^. The *g*_iso_ value of [Cp_2_Co][U^VI^O_2_Cl(**L**^**F•**^)] was similar at 1.9893, and the shape of the signal is consistent with the carbon radical.^11^ The observation of this EPR signal suggests that the formation of the isolated dimer uranyl(V) complex proceeds *via* the one-electron reduction of the ligand and thus through the formation of a ligand radical complex [U^VI^O_2_Cl(**L**^**Mes•**^)]^−^.

The *g*-value is slightly lower in comparison to that of the free electron, which is due to the interaction of the unpaired spin with the larger spin–orbital coupling associated with the uranium nucleus. Other reported U^VI^–(**L**^**•**^) species display similar *g*-values and line broadening in their fluid solution EPR spectra.^11,23^ In each case, the unpaired spin was assigned to the ligand moiety, with the low *g*-values due to spin–orbital coupling to the uranium center.^3^

### DFT Calculations

**Figure 8:**
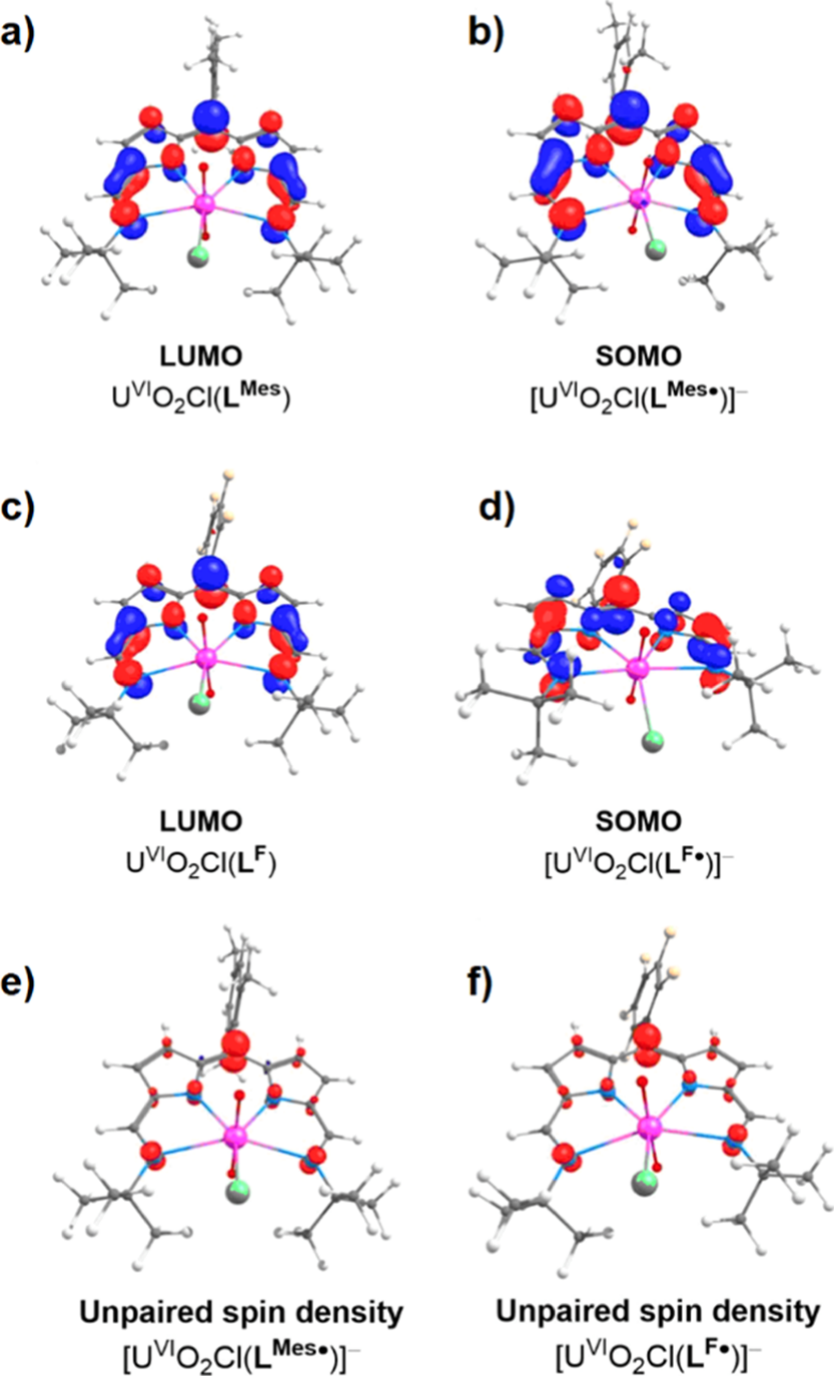
Molecular orbital plots of U^VI^O_2_Cl(**L**^**Mes**^) and [U^VI^O_2_Cl(**L**^**Mes•**^)]^−^ (a,b) and U^VI^O_2_Cl(**L**^**F**^) and [U^VI^O_2_Cl(**L**^**F•**^)]^−^ (c,d) and spin density plots of the singly reduced complexes [U^VI^O_2_Cl(**L**^**•**^)]^−^ (e,f). The ISO value is 0.02 au. Positive is blue; negative is red.

A variety of DFT calculations were undertaken on both U^VI^O_2_Cl(**L**^**Mes**^) and U^VI^O_2_Cl(**L**^**F**^) and reveal that for both the cases, the LUMOs are located entirely on the ligand, indicating that the incorporation of the electron-donating *meso*-mesityl substituent does not modify the molecular orbitals to a great extent (Figure 8). In addition, the singly occupied molecular orbitals of [U^VI^O_2_Cl(**L**^**Mes•**^)]^−^ and [U^VI^O_2_Cl(**L**^**F•**^)]^−^ are also ligand-based, and the unpaired spin density maps of both show that the electron density is located on the ligand, primarily on the *meso*-carbon, and not on the uranium atom; this further confirms the radical character of the ligand after one-electron reduction.

This reduction process is supported by the solid-state structure obtained for [Cp_2_Co][U^VI^O_2_Cl(**L**^**F•**^)].^11^ However, it is clear that from the experimental reduction of the *meso*-mesityl complex U^VI^O_2_Cl(**L**^**Mes**^), only the uranyl(V) dimer [U^V^O_2_(**L**^**Mes**^)]_2_ is obtained, which is not rationalized through this ligand reduction process.

**Scheme 4:**
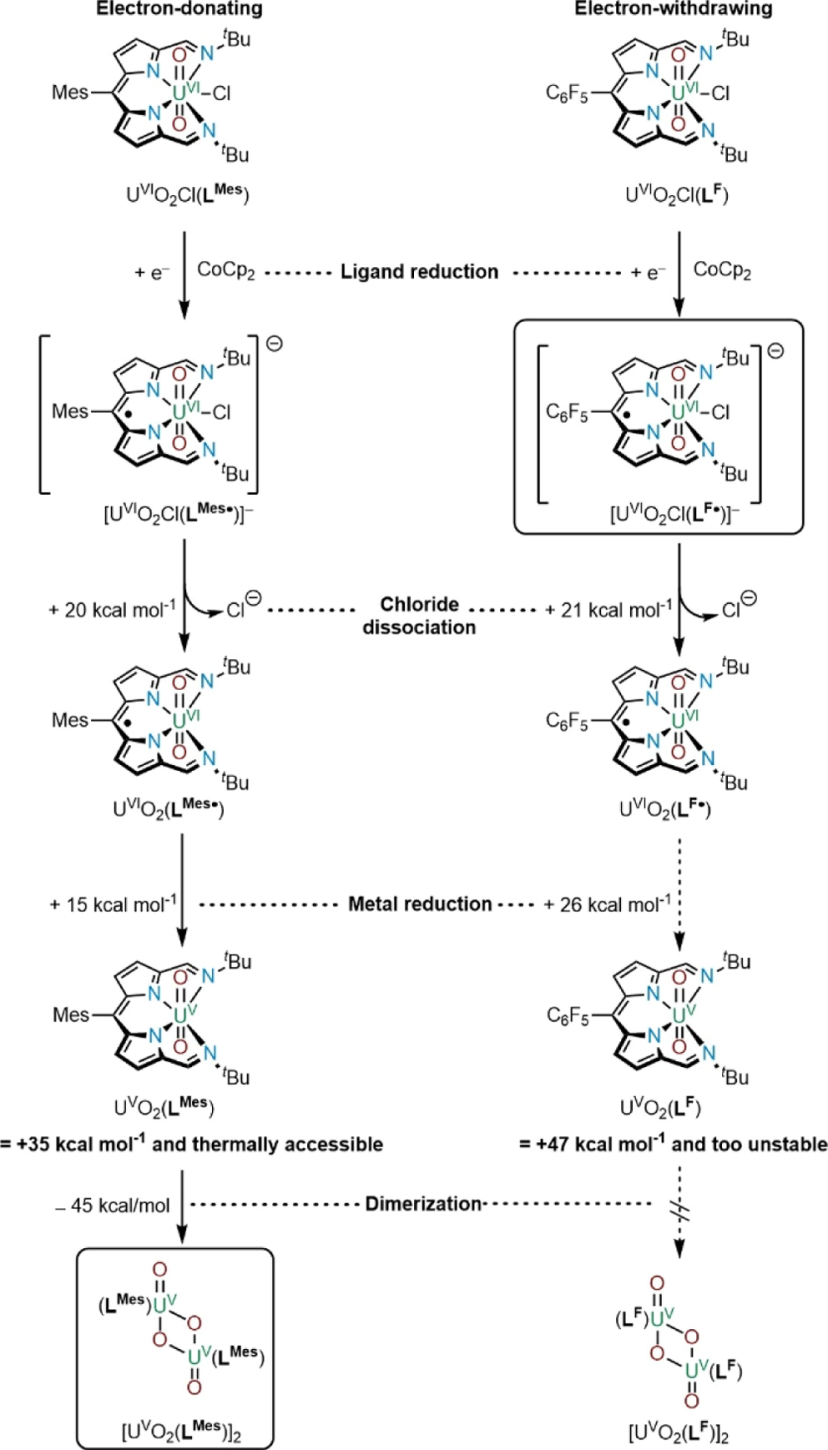
Reduction Processes for U^VI^O_2_Cl(**L**^**Mes**^) and U^VI^O_2_Cl(**L**^**F**^) Resulting in [U^V^O_2_(**L**^**Mes**^)]_2_ and [Cp_2_Co][U^VI^O_2_CL(**L**^**F•**^)], Respectively

The similarity in the CVs of U^VI^O_2_Cl(**L**^**Mes**^) and the cationic uranyl complex [U^VI^O_2_(**L**^**F**^)][BAr^F^] suggests that reduction is in concert with chloride dissociation (Scheme 4). This step was computed and results in the formation of U^VI^O_2_(**L**^**Mes•**^) and U^VI^O_2_(**L**^**F•**^), which are energetically plausible for both complexes at +20 kcal mol^–1^ for U^VI^O_2_(**L**^**Mes•**^) and +21 kcal mol^–1^ for U^VI^O_2_(**L**^**F•**^). In both cases, electron transfer from the ligand to the metal does not occur.

The next computed step involves the formation of a U^V^–(**L**) monomer through electron transfer from the ligand to the metal, thus forming U^V^O_2_(**L**^**Mes**^) and U^V^O_2_(**L**^**F**^), respectively. This step is estimated to cost +15 kcal mol^–1^ for the U^V^O_2_(**L**^**Mes**^) and +26 kcal mol^–1^ for U^V^O_2_(**L**^**F**^). Therefore, the former formation is thermally accessible at +35 kcal mol^–1^ from U^VI^O_2_Cl(**L**^**Mes**^) but inaccessible for U^V^O_2_(**L**^**F**^) at +47 kcal mol^–1^. It is important to note that dimerization of the monomeric uranyl(V) complex U^V^O_2_(**L**^**Mes**^) to form the uranyl(V) dimer [U^V^O_2_(**L**^**Mes**^)]_2_ is exothermic by −45 kcal mol^–1^, making the whole process exothermic by 10 kcal mol^–1^.

## Discussion

Changing the meso substituent of the dipyrrin ligand in the uranyl complexes U^VI^O_2_Cl(**L**) from electron-withdrawing **L**^**F**^ to electron-donating **L**^**Mes**^ modifies the stability of the products formed upon single-electron reduction; for **L**^**F**^, the ligand-reduced complex U^VI^O_2_Cl(**L**^**F•**^) is isolated, whereas in contrast, for **L**^**Mes**^, the uranyl(V) dimer [U^V^O_2_(**L**^**Mes**^)]_2_ is seen. This difference in the reduction product is also implied experimentally from the differences in the CVs of these complexes and the instability of the singly reduced complex [U^VI^O_2_Cl(**L**^**Mes•**^)]^−^ by EPR spectroscopy.

The difference in reactivity caused by the mesityl meso substituent becomes clear when analyzing the different steps of the reduction processes computationally. Although both U^VI^O_2_Cl(**L**^**Mes**^) and U^VI^O_2_Cl(**L**^**F**^) can form the chloride-free ligand–radical complexes U^VI^O_2_(**L**^**Mes•**^) and U^VI^O_2_(**L**^**F•**^), respectively, only the mesityl analogue undergoes an electron transfer from the ligand to the metal. The latter process requires an increase in energy of 11 kcal mol^–1^ for the pentafluorophenyl analogue, making it thermally inaccessible. It is therefore shown that the pentafluorophenyl substituent stabilizes the ligand–radical complex, causing electron transfer to the metal to be less favorable, whereas the mesityl substituent destabilizes the ligand–radical complexes, facilitating the electron transfer. Once the U^V^O_2_(**L**^**Mes**^) monomer is formed, the formation of the diamond-shaped, oxo-bridged uranyl(V) dimer is facile and is promoted by the increased Lewis basicity of the axial oxos of the reduced uranyl center.^24^

## Conclusions

We have shown that the variation of the meso substituent in uranyl Schiff-base dipyrrin complexes moderates the stabilities of the neutral, ligand-reduced complexes U^VI^O_2_(**L**^**•**^), which affects the subsequent electron transfer to the metal. It is anticipated that further modification of the dipyrrin ligand, for example, increasing the steric bulk at the α-positions of the pyrrole or substituting at the β-positions, could lead to the formation of new uranyl(V) products by suppressing dimerization. Furthermore, the facile ligand modifications described here may prove important in the design of future reactions such as electron transfer or oxo-atom transfer in which controlled access to either the ligand radical or uranyl(V) complexes is desired.

## Supporting Information Available

Additional synthetic procedures, X-ray crystallography, DFT calculations, EPR spectroscopy, and electrochemical methods (PDF)
