# Supplementary material for: Role of the Meso Substituent in Defining the Reduction of Uranyl Dipyrrin Complexes
Source: Inorg Chem. 2022 Dec 6;61(50):20424–32. doi: 10.1021/acs.inorgchem.2c03048 (PMC9768749; doi:10.1021/acs.inorgchem.2c03048)
Supplement: Supplementary file 1 — ic2c03048_si_001.pdf [file ic2c03048_si_001.pdf]

# The role of the *meso*-substituent in defining the reduction of uranyl dipyrrin complexes

Karlotta van Rees[a], Thayalan Rajeshkumar[b], Laurent Maron[b], Stephen Sproules[c], and Jason B. Love*[a]

[a]EaStCHEM School of Chemistry, University of Edinburgh, Joseph Black Building, David Brewster Road, Edinburgh, EH9 3FJ; [b]LPCNO, INSA, Uiversité de Toulouse, 135, avenue de Rangueil, 31077, Toulouse cedex 4, France; [c]WestCHEM School of Chemistry, University of Glasgow, Glasgow, G12 8QQ, UK.

## Contents

**1 General procedures** ........ 2

**2 Synthesis of $U^{VI}O_2Cl(L^{Mes})$** ........ 4

**3 Crystallography** ........ 5

**4 Electrochemistry** ........ 9

**5 EPR Spectroscopy** ........ 10

**6 DFT calculations** ........ 11

**6.1 Molecular Orbitals** ........ 11

**6.2 Structural parameters and energies** ........ 12

**6.3 Optimized coordinates with dispersion correction** ........ 15

**7 References** ........ 27

# 1 General procedures

**Caution:** *Depleted uranium (primary isotope $^{238}U$) is a weak α-emitter (4.197 MeV) with a half-life of 4.47×109 years. Manipulations and reactions should be carried out in monitored fume hoods or in an inert atmosphere glovebox in a radiation laboratory equipped with α- and β-counting equipment.*

The syntheses of all air- and moisture-sensitive compounds were carried out using standard Schlenk techniques under an atmosphere of dry argon. Vacuum Atmospheres and MBraun glove boxes were used to manipulate and store air- and moisture-sensitive compounds under an atmosphere of dried and deoxygenated dinitrogen. The solvents benzene-$d_6$ and pyridine-$d_5$ were refluxed over potassium metal overnight, trap-to-trap distilled and three times free-pump-thaw degassed prior to use. All glassware was dried in an oven at 160 °C, cooled under $10^{-3}$ mbar vacuum and then purged with argon. Prior to use, all Fisherbrand R 1.2 mm retention glass microfiber filters and stainless-steel cannula were dried in an oven at 160 °C overnight. All solvents for use with air- and moisture-sensitive compounds were stored in Teflon-tapped ampoules containing pre-dried 4 Å molecular sieves. Solvents were collected from a solvent purification system (Innovation Technologies), where they had been passed over a column of molecular sieves for 24 hours prior to collection. They were then degassed prior to use and subsequent storage. All chemicals were used as used as received without any purification, unless otherwise specified. Tetrabutylammonium hexafluorophosphate, $[^nBu_4N][PF_6]$, was recrystallized twice from absolute ethanol and further dried for two days under vacuum.

$^1H$ NMR spectra were recorded on a Bruker AVA400 spectrometer operating at 399.90 MHz, a Bruker AVA500 or Bruker PRO500 operating at 500.12 MHz or a Bruker AVA600 spectrometer operating at 599.81 MHz. $^{13}C\{^1H\}$ NMR spectra were recorded on a Bruker AVA500 or Bruker PRO500 operating at 125.76 MHz. $^{19}F\{^1H\}$ NMR spectra were recorded on a Bruker AVA500 spectrometer operating at 470.59 MHz. Chemical shifts are reported in parts per million (ppm). $^1H$ and $^{13}C\{^1H\}$ NMR spectra are referenced to residual solvent resonances calibrated against an external standard, $SiMe_4$ (d = 0 ppm). $^{19}F\{^1H\}$ NMR spectra are referenced to an external standard, $CCl_3F$ (d = 0 ppm). All spectra were recorded at 298 K unless otherwise specified. All data were processed using MestReNova 12.0.3. Full assignment in the supplementary information.

Single crystal X-ray diffraction data were collected at 120 K on an Oxford Diffraction Excalibur diffractometer using graphite monochromated Mo-Ka radiation equipped with an Eos CCD detector (λ = 0.71073 Å), or at 120 K on a Supernova, Dual, Cu at Zero Atlas diffractometer using Cu-Kalpha radiation (λ = 1.5418 Å). Structures were solved using ShelXT direct methods or intrinsic phasing and refined using a full-matrix least-square refinement on $|F|^2$ using ShelXL.[1-3] All programs were used within the Olex suite.[4] All non-hydrogen atoms refined with anisotropic displacement parameters and H-parameters were constrained to parent atoms and refined using a riding model unless otherwise specified. All X-ray crystal structures were analyzed and illustrated using Mercury 4.3.1.

Elemental analyses were carried out by Mr Stephen Boyer at the London Metropolitan University and Elemental Microanalysis Ltd., measured in duplicate. All FT-IR spectra were recorded using JASCO 410 or JASCO 460 plus spectrometers. Intensities are assigned as: w = weak, m = medium, and s = strong. All UV-vis absorption spectra

were recorded on a Jasco V-670 spectrometer on a 10 mm quartz cuvette, fitted with a septum for air-sensitive compounds.

The optimization of different spin states for uranium complexes was carried out by employing DFT hybrid functional (B3PW91)[5-6] along with small core pseudopotential Stuggart basis set for uranium, chlorine with additional polarization functions for chlorine atom.[7-8] Pople basis sets (6-31G** for carbon, nitrogen, oxygen, hydrogen atoms) were employed for the rest of the atoms.[9-10] Frequency calculations were performed to locate minima for the optimized structures. Disperison corrections were included in our calculations by employing D3 version of Grimme's dispersion with Becke-Johnson damping.[11] All the calculations were performed using Gaussian 09 suite of programs.[12]

# 2 Synthesis of $U^{VI}O_2Cl(L^{Mes})$

**Method B:** The synthesis was conducted under an inert atmosphere. A mixture of $[U^{VI}O_2\{N(SiMe_3)_2\}_2(THF)_2]$ (22 mg, 0.03 mmol, 0.5 eq) and $[U^{VI}O_2Cl_2(THF)_2]$ (14.5 mg, 0.03 mmol, 0.5 eq) was suspended in $C_6D_6$ (5 mL) and stirred for 15 min resulting in a dark orange suspension which was dropwise added to a dark orange-brown solution of H$\mathbf{L}^{\mathbf{Mes}}$ (25 mg, 0.06 mmol, 1 eq) in $C_6D_6$ (2 mL). The solution turned dark red and $^1$H NMR spectroscopic analysis after 1 h confirmed the formation of ~40 % $U^{VI}O_2\{N(SiMe_3)_2\}(\mathbf{L}^{\mathbf{Mes}})$ and ~10 % $U^{VI}O_2Cl(\mathbf{L}^{\mathbf{Mes}})$. After stirring the solution for 16 h the solution was pink of color and the $^1$H NMR showed the formation of a new species in ~50 % $U^{VI}O_2Cl(\mathbf{L}^{\mathbf{Mes}})$ (by $^1$H NMR integration). The reaction was left to stir over the weekend and this resulted in the full conversion of H$\mathbf{L}^{\mathbf{Mes}}$ into $U^{VI}O_2Cl(\mathbf{L}^{\mathbf{Mes}})$, indicative by the purple color of the solution.

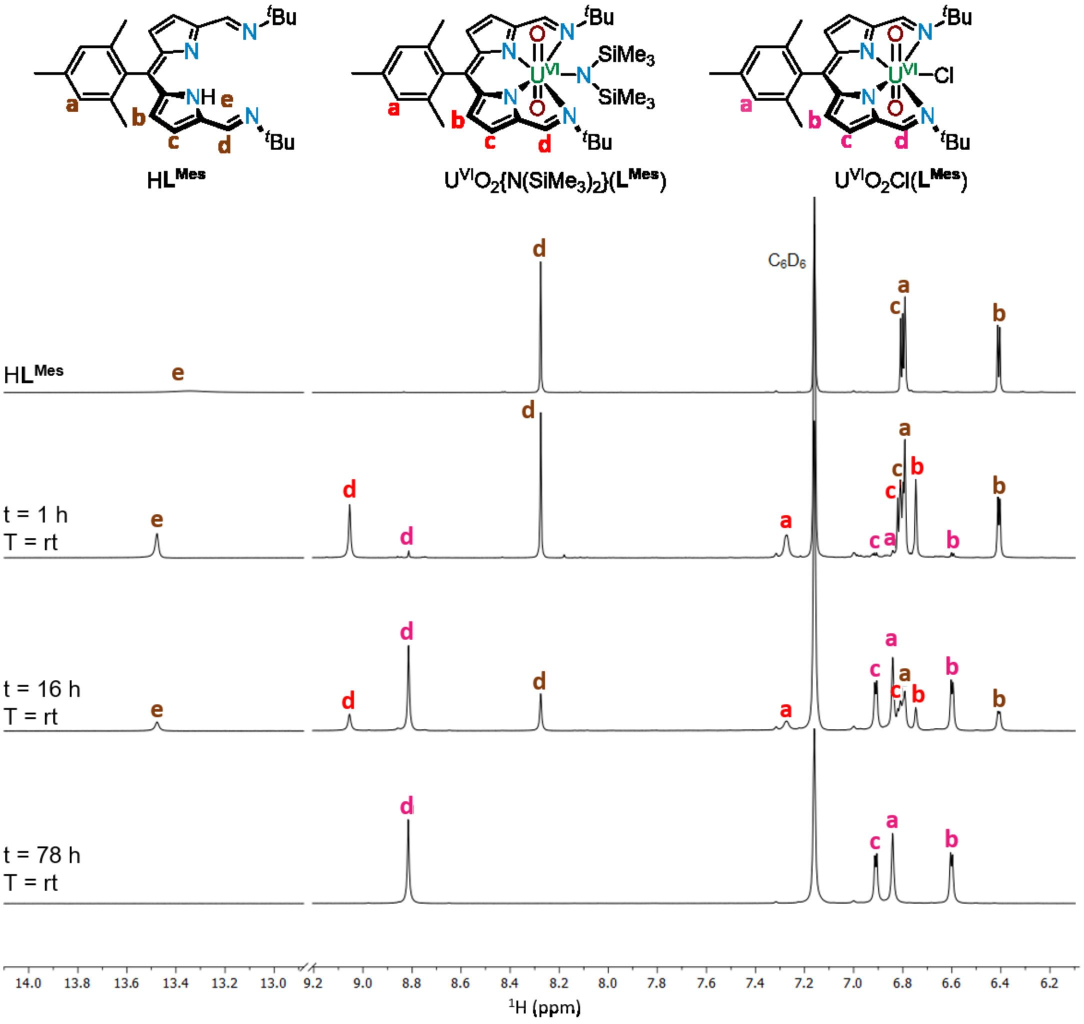


**Figure S1:** Stacked low-field regions of the $^1$H NMR spectra in benzene-$d_6$. The first row is the compound H$\mathbf{L}^{\mathbf{Mes}}$, whereas the other rows are aliquots taken during the synthesis of $U^{VI}O_2Cl(\mathbf{L}^{\mathbf{Mes}})$ via method B.

# 3 Crystallography

**Table S1** Crystal data for H**L**$^{\mathbf{Mes}}$. CCDC: 2201753.

| | |
|---|---|
| **Crystal data** | |
| Chemical formula | $C_{28}H_{36}N_4$ |
| $M_r$ | 428.61 |
| Crystal system, space group | Triclinic, *P*1 |
| Temperature (K) | 120 |
| *a*, *b*, *c* (Å) | 12.9528 (16), 13.507 (2), 16.0619 (14) |
| α, β, γ (°) | 90.431 (9), 107.889 (9), 105.060 (12) |
| *V* (Å$^3$) | 2570.7 (6) |
| *Z* | 4 |
| Radiation type | Mo *K*α |
| μ (mm$^{-1}$) | 0.07 |
| Crystal size (mm) | 0.26 × 0.22 × 0.13 |
| **Data collection** | |
| Diffractometer | Xcalibur, Eos |
| Absorption correction | Analytical<br>*CrysAlis PRO* 1.171.40.53 (Rigaku Oxford Diffraction, 2019) Analytical numeric absorption correction using a multifaceted crystal model based on expressions derived by R.C. Clark & J.S. Reid. (Clark, R. C. & Reid, J. S. (1995). Acta Cryst. A51, 887-897) Empirical absorption correction using spherical harmonics, implemented in SCALE3 ABSPACK scaling algorithm. |
| $T_{min}$, $T_{max}$ | 0.994, 0.998 |
| No. of measured, independent and observed [$I > 2\sigma(I)$] reflections | 64383, 5347, 4281 |
| $R_{int}$ | 0.177 |
| $\theta_{max}$ (°) | 20.8 |
| $(\sin \theta/\lambda)_{max}$ (Å$^{-1}$) | 0.500 |
| **Refinement** | |
| $R[F^2 > 2\sigma(F^2)]$, $wR(F^2)$, $S$ | 0.134, 0.245, 1.30 |
| No. of reflections | 5347 |
| No. of parameters | 603 |
| H-atom treatment | H atoms treated by a mixture of independent and constrained refinement |
| $\Delta\rho_{max}$, $\Delta\rho_{min}$ (e Å$^{-3}$) | 0.26, −0.23 |

**Table S2** Crystal data for K(**L**$^{\mathbf{Mes}}$). CCDC: 2201750.

| | |
|---|---|
| **Crystal data** | |
| Chemical formula | $C_{36}H_{51}KN_4O_2$ |
| $M_r$ | 610.90 |
| Crystal system, space group | Triclinic, *P*1 |
| Temperature (K) | 120 |
| *a*, *b*, *c* (Å) | 13.9231 (7), 14.6703 (8), 18.5249 (10) |
| α, β, γ (°) | 76.530 (5), 85.807 (4), 73.742 (4) |
| *V* (Å$^3$) | 3532.5 (3) |
| *Z* | 4 |
| Radiation type | Mo *K*α |
| μ (mm$^{-1}$) | 0.19 |
| Crystal size (mm) | 0.78 × 0.28 × 0.10 |
| **Data collection** | |
| Diffractometer | Xcalibur, Eos |
| Absorption correction | Analytical<br>*CrysAlis PRO* 1.171.40.53 (Rigaku Oxford Diffraction, 2019) Analytical numeric absorption correction using a multifaceted crystal model based on expressions derived by R.C. Clark & J.S. Reid. (Clark, R. C. & Reid, J. S. (1995). Acta Cryst. A51, 887-897) Empirical absorption correction using spherical harmonics, implemented in SCALE3 ABSPACK scaling algorithm. |
| $T_{min}$, $T_{max}$ | 0.980, 0.996 |
| No. of measured, independent and observed [$I > 2\sigma(I)$] reflections | 27303, 27303, 13930 |
| $R_{int}$ | Twin data |
| $(\sin \theta/\lambda)_{max}$ (Å$^{-1}$) | 0.690 |
| **Refinement** | |
| $R[F^2 > 2\sigma(F^2)]$, $wR(F^2)$, $S$ | 0.090, 0.169, 0.96 |
| No. of reflections | 27303 |
| No. of parameters | 794 |
| H-atom treatment | H-atom parameters constrained |
| $\Delta\rho_{max}$, $\Delta\rho_{min}$ (e Å$^{-3}$) | 0.61, −0.51 |

**Table S3.** Crystal data for $U^{VI}O_2Cl$(**L$^{Mes}$**). CCDC: 2201751.

| | |
|---|---|
| **Crystal data** | |
| Chemical formula | $C_{28}H_{35}ClN_4O_2U$ |
| $M_r$ | 733.08 |
| Crystal system, space group | Monoclinic, $P2_1/c$ |
| Temperature (K) | 120 |
| *a*, *b*, *c* (Å) | 20.6877 (2), 8.4433 (1), 15.8930 (2) |
| α, β, γ (°) | 90, 91.050 (1), 90 |
| *V* (Å$^3$) | 2775.60 (5) |
| *Z* | 4 |
| Radiation type | Mo *K*α |
| μ (mm$^{-1}$) | 5.98 |
| Crystal size (mm) | 0.24 × 0.17 × 0.10 |
| **Data collection** | |
| Diffractometer | Xcalibur, Eos |
| Absorption correction | Analytical<br>*CrysAlis PRO* 1.171.40.53 (Rigaku Oxford Diffraction, 2019) Analytical numeric absorption correction using a multifaceted crystal model based on expressions derived by R.C. Clark & J.S. Reid. (Clark, R. C. & Reid, J. S. (1995). Acta Cryst. A51, 887-897) Empirical absorption correction using spherical harmonics, implemented in SCALE3 ABSPACK scaling algorithm. |
| $T_{min}$, $T_{max}$ | 0.593, 0.770 |
| No. of measured, independent and observed [$I > 2\sigma(I)$] reflections | 110279, 5664, 5352 |
| $R_{int}$ | 0.037 |
| $(\sin \theta/\lambda)_{max}$ (Å$^{-1}$) | 0.625 |
| **Refinement** | |
| $R[F^2 > 2\sigma(F^2)]$, $wR(F^2)$, $S$ | 0.016, 0.035, 1.12 |
| No. of reflections | 5664 |
| No. of parameters | 334 |
| H-atom treatment | H-atom parameters constrained |
| $\Delta\rho_{max}$, $\Delta\rho_{min}$ (e Å$^{-3}$) | 0.59, −0.99 |

**Table S4.** Crystal data for $[U^{V}O_2(\mathbf{L^{Mes}})]_2$. CCDC: 2201752.

| | |
|---|---|
| **Crystal data** | |
| Chemical formula | $2(C_{56}H_{70}N_8O_4U_2)\cdot C_6H_6$ |
| $M_r$ | 2868.62 |
| Crystal system, space group | Triclinic, *P*1 |
| Temperature (K) | 120 |
| *a*, *b*, *c* (Å) | 12.7988 (2), 15.2792 (4), 16.0886 (3) |
| α, β, γ (°) | 85.884 (2), 89.630 (1), 82.270 (2) |
| *V* (Å$^3$) | 3109.57 (11) |
| *Z* | 1 |
| Radiation type | Mo *K*α |
| μ (mm$^{-1}$) | 5.25 |
| Crystal size (mm) | 0.13 × 0.10 × 0.04 |
| **Data collection** | |
| Diffractometer | Xcalibur, Eos |
| Absorption correction | Analytical<br>*CrysAlis PRO* 1.171.40.53 (Rigaku Oxford Diffraction, 2019) Analytical numeric absorption correction using a multifaceted crystal model based on expressions derived by R.C. Clark & J.S. Reid. (Clark, R. C. & Reid, J. S. (1995). Acta Cryst. A51, 887-897) Empirical absorption correction using spherical harmonics, implemented in SCALE3 ABSPACK scaling algorithm. |
| $T_{min}$, $T_{max}$ | 0.587, 0.829 |
| No. of measured, independent and observed [$I > 2\sigma(I)$] reflections | 118303, 11381, 9582 |
| $R_{int}$ | 0.074 |
| $(\sin \theta/\lambda)_{max}$ (Å$^{-1}$) | 0.602 |
| **Refinement** | |
| $R[F^2 > 2\sigma(F^2)]$, $wR(F^2)$, $S$ | 0.032, 0.059, 1.03 |
| No. of reflections | 11381 |
| No. of parameters | 676 |
| H-atom treatment | H-atom parameters constrained |
| $\Delta\rho_{max}$, $\Delta\rho_{min}$ (e Å$^{-3}$) | 1.12, −0.87 |

# 4 Electrochemistry

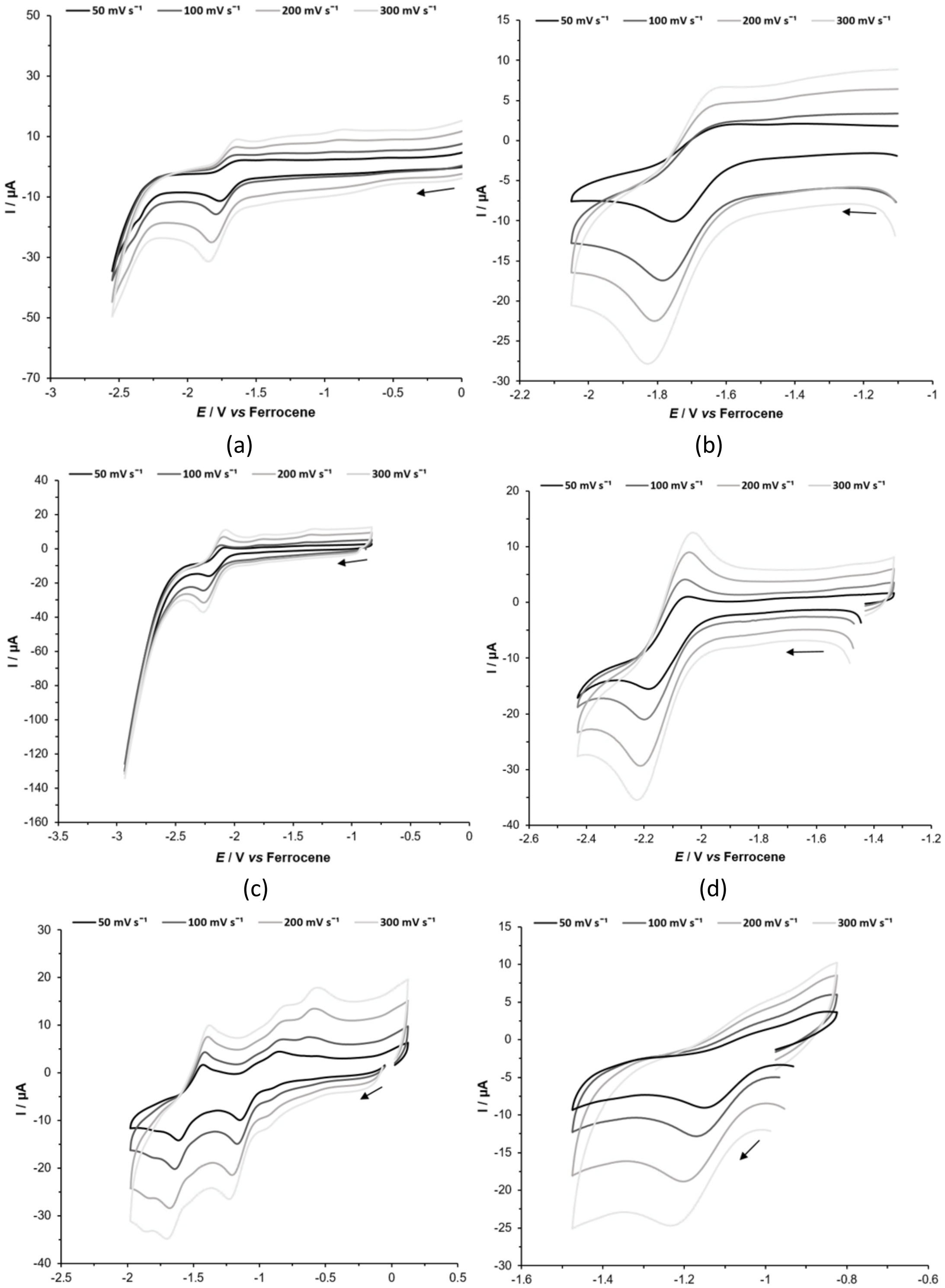


**Figure S2:** Cyclic voltammograms of H**L**$^{\mathbf{Mes}}$, K(**L**$^{\mathbf{Mes}}$), and $U^{VI}O_2Cl$(**L**$^{\mathbf{Mes}}$). H**L**$^{\mathbf{Mes}}$: (a) variable scan rates and (b) isolated reversible reduction I at variable scan rates. K(**L**$^{\mathbf{Mes}}$): (c) variable scan rates and (d) isolated reversible reduction I at variable scan rates. $U^{VI}O_2Cl$(**L**$^{\mathbf{Mes}}$): (e) variable scan rates and (f) isolated irreversible reduction I at variable scan rates. All were measured at 1 mM $CH_2Cl_2$ solutions (0.1 M $[^nBu_4N][PF_6]$ supporting electrolyte, glassy-carbon working electrode, platinum gauze counter electrode, and silver wire quasi-reference electrode). Potentials are references against the $Fc/Fc^+$ couple recorded under identical conditions.

# 5 EPR Spectroscopy

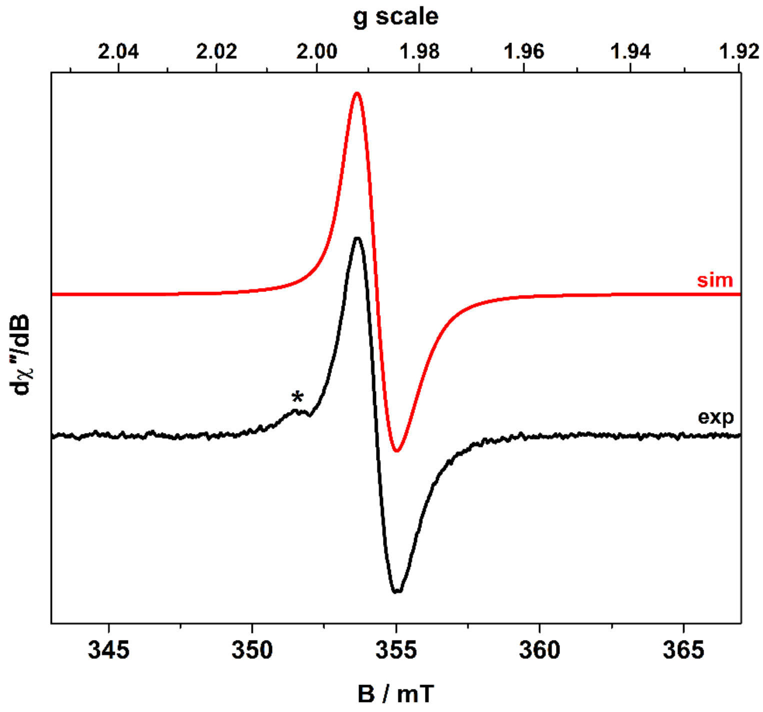


**Figure S3:** X-band EPR spectrum of $[U^{VI}O_2Cl(\mathbf{L}^{Mes\bullet})]^-$ generated in $CH_2Cl_2$ solution at ambient temperature (experimental conditions: frequency, 9.8606 GHz; power, 2.0 mW; modulation, 0.2 mT). Experimental data are shown by the black line, and simulation by the dashed red trace: $g_{iso}$ = 1.987. The asterisk denoted trace $(\mathbf{L}^{Mes\bullet})^-$ in solution formed from $H\mathbf{L}^{Mes}$ present as a baseline impurity. The impurity is visible in the $^1H$ NMR spectrum in Figure S4.

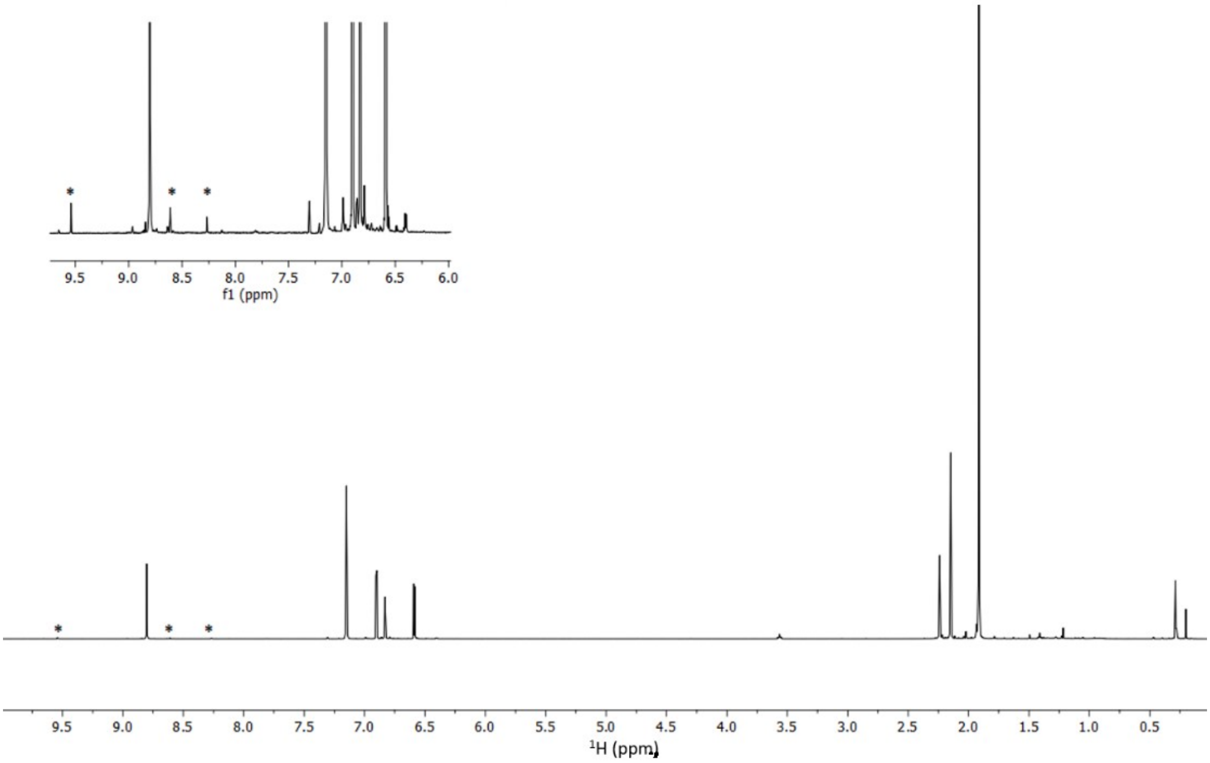


**Figure S4:** $^1H$ NMR spectrum of $U^{VI}O_2Cl(\mathbf{L}^{Mes})$ in benzene-$d_6$ showing the minor $H\mathbf{L}^{Mes}$ compound as a baseline impurity denoted with an asterisk.

# 6 DFT calculations

## 6.1 Molecular Orbitals

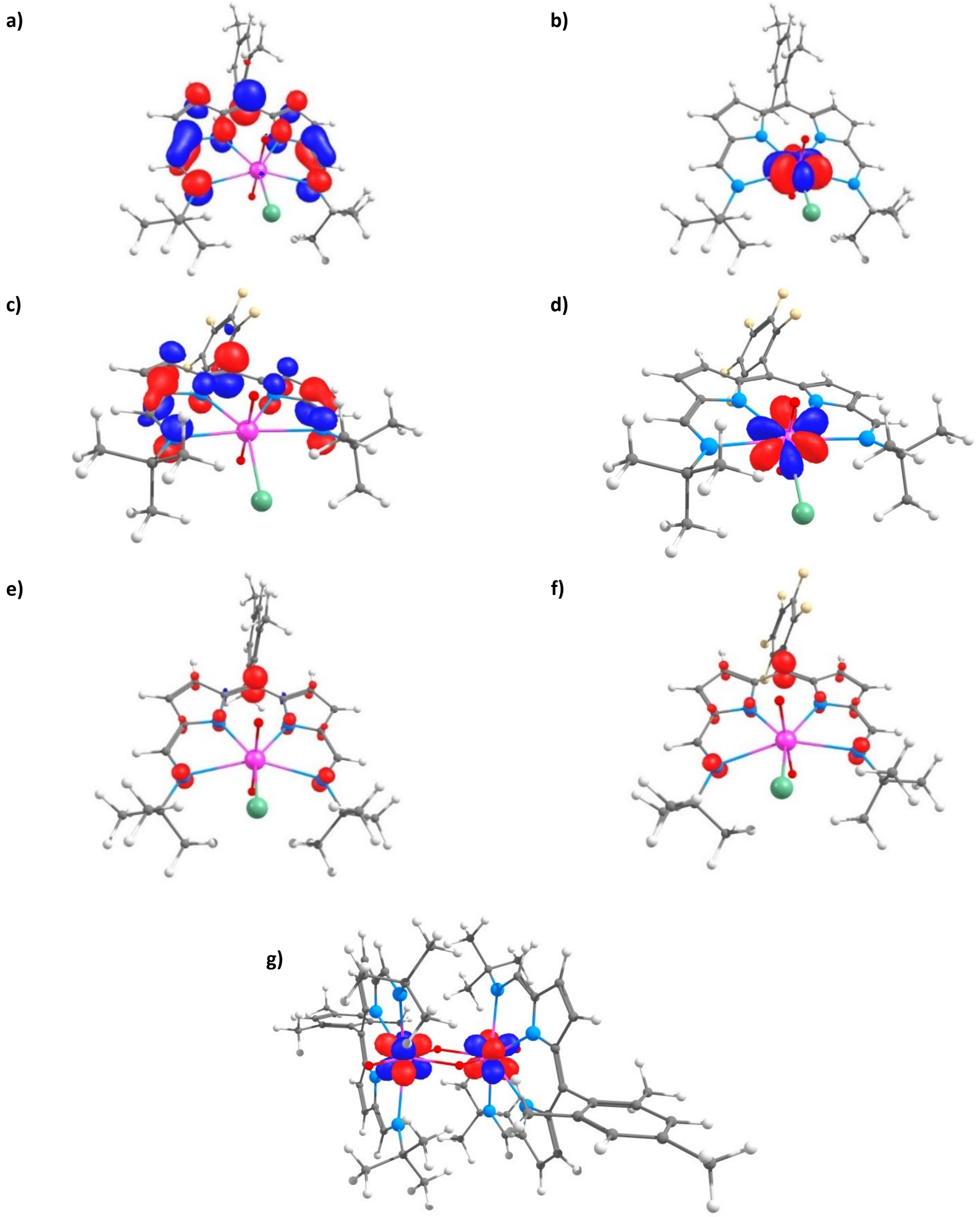


**Figure S5**: DFT computed Molecular orbitals: (a) HOMO of $[U^{VI}O_2Cl(\mathbf{L^{Mes}})]^-$ (b) LUMO of $[U^{VI}O_2Cl(\mathbf{L^{Mes}})]^-$ (c) HOMO of $[U^{VI}O_2Cl(\mathbf{L^{F}})]^-$ (d) LUMO of $[U^{VI}O_2Cl(\mathbf{L^{F}})]^-$ (e) spin density plot of $[U^{VI}O_2Cl(\mathbf{L^{Mes}})]^-$ (f) spin density plot of $[U^{VI}O_2Cl(\mathbf{L^{F}})]^-$ (g) HOMO of $[U^{V}O_2(\mathbf{L^{Mes}})]_2$. The ISO value is 0.02 au. Positive is blue; negative is red.

## 6.2 Structural parameters and energies

**Table S5:** Selected structural parameters for $U^{VI}O_2Cl$(**L**$^{F}$) and $U^{VI}O_2Cl$(**L**$^{Mes}$). Bond distance in Å and angles in degrees.

| Bond | $U^{VI}O_2Cl(L^F)$ | | | | $U^{VI}O_2Cl(L^{Mes})$ |
|---|---|---|---|---|---|
| | DFT (gas) | DFT+disp (gas) | DFT (solv) | X-ray | DFT+disp (gas) |
| U1–Cl2 | 2.65 | 2.65 | 2.69 | 2.69 | 2.65 |
| U1–O3 | 1.76 | 1.76 | 1.76 | 1.77 | 1.75 |
| U1–O4 | 1.75 | 1.75 | 1.76 | 1.76 | 1.76 |
| U1–N5 | 2.78 | 2.73 | 2.77 | 2.67 | 2.73 |
| U1–N6 | 2.52 | 2.50 | 2.50 | 2.48 | 2.51 |
| U1–N7 | 2.78 | 2.73 | 2.76 | 2.68 | 2.72 |
| U1–N8 | 2.52 | 2.50 | 2.50 | 2.47 | 2.51 |
| **Angle** | | | | | |
| O3–U1–O4 | 177.5 | 176.9 | 177.5 | 176.2 | 176.8 |

**Table S6:** Selected structural parameters for $[U^{VI}O_2Cl$(**L**$^{F\bullet}$)$]^{-}$. Bond distance in Å and angles in degrees.

| Bond | DFT (gas) | DFT+disp (gas) |
|---|---|---|
| U1–Cl2 | 2.74 | 2.74 |
| U1–O3 | 1.76 | 1.76 |
| U1–O4 | 1.76 | 1.76 |
| U1–N5 | 2.77 | 2.71 |
| U1–N6 | 2.48 | 2.47 |
| U1–N7 | 2.76 | 2.70 |
| U1–N8 | 2.48 | 2.47 |
| **Angle** | | |
| O3–U1–O4 | 179.3 | 178.6 |

**Table S7:** Selected structural parameters for $[U^{VI}O_2Cl$(**L**$^{Mes\bullet}$)$]^{-}$. Bond distance in Å and angles in degrees.

| Bond | DFT (gas) | DFT+disp (gas) |
|---|---|---|
| U62–Cl61 | 2.73 | 2.73 |
| U62–O54 | 1.76 | 1.76 |
| U62–O55 | 1.76 | 1.76 |
| U1–N50 | 2.77 | 2.71 |
| U1–N51 | 2.48 | 2.48 |
| U1–N52 | 2.49 | 2.48 |
| U1–N53 | 2.75 | 2.69 |
| **Angle** | | |
| O3–U1–O4 | 179.1 | 178.4 |

**Table S8:** Selected structural parameters for $[U^{V}O_2(\mathbf{L^F})]_2$ and $[U^{V}O_2(\mathbf{L^{Mes}})]_2$. Bond distance in Å and angles in degrees.

| Bond | $[U^{V}O_2(L^F)]_2$ | | | $[U^{V}O_2(L^{Mes})]_2$ |
|---|---|---|---|---|
| | DFT (gas) | DFT+disp (gas) | X-ray | DFT+disp (gas) |
| U1–O2 | 1.90 | 1.91 | 1.94 | 1.90 |
| U1–O3 | 1.80 | 1.81 | 1.83 | 1.80 |
| U1–N4 | 2.55 | 2.52 | 2.50 | 2.51 |
| U1–N5 | 2.55 | 2.52 | 2.49 | 2.52 |
| U1–N6 | 2.73 | 2.66 | 2.67 | 2.66 |
| U1–N7 | 2.74 | 2.67 | 2.69 | 2.64 |
| U71–O72 | 1.90 | 1.91 | 1.94 | 1.90 |
| U71–O73 | 1.80 | 1.81 | 1.83 | 1.80 |
| U71–N74 | 2.55 | 2.53 | 2.50 | 2.51 |
| U71–N75 | 2.55 | 2.52 | 2.49 | 2.52 |
| U71–N76 | 2.73 | 2.66 | 2.67 | 2.66 |
| U71–N77 | 2.74 | 2.68 | 2.69 | 2.64 |
| **Angle** | | | | |
| O2–U1–O3 | 179.04 | 179.17 | 175.20 | 179.50 |
| O72–U71–O73 | 179.04 | 179.11 | 175.20 | 179.50 |

**Table S9:** Computed enthalpy (H) values for DFT optimised structures.

| Complex | H (Hatrees) |
|---|---|
| $U^{VI}O_2Cl(\mathbf{L^F})$ | –1948.984780 |
| $[U^{VI}O_2Cl(\mathbf{L^{F\bullet}})]^-$ | –1949.061566 |
| $U^{VI}O_2(\mathbf{L^{F\bullet}})$ | –1933.903666 |
| $U^{V}O_2(\mathbf{L^F})$ | –1933.871793 |
| $[U^{V}O_2(\mathbf{L^F})]_2$ | –3867.878659 |
| $U^{VI}O_2Cl(\mathbf{L^{Mes}})$ | –2327.108522 |
| $[U^{VI}O_2Cl(\mathbf{L^{Mes\bullet}})]^-$ | –2327.196158 |
| $U^{VI}O_2(\mathbf{L^{Mes\bullet}})$ | –2312.038236 |
| $U^{V}O_2(\mathbf{L^{Mes}})$ | –2311.996802 |
| $[U^{V}O_2(\mathbf{L^{Mes}})]_2$ | –4624.127531 |

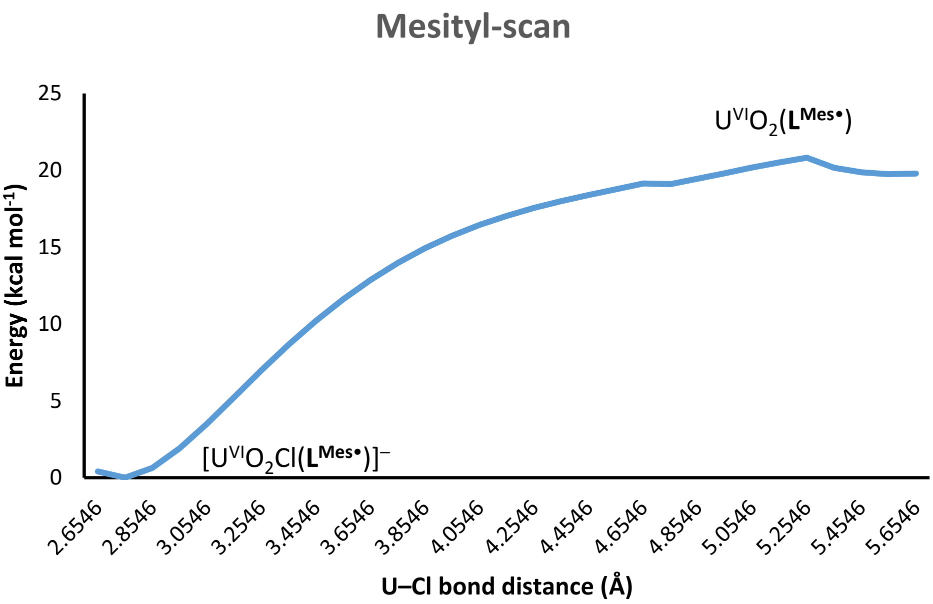


(a)

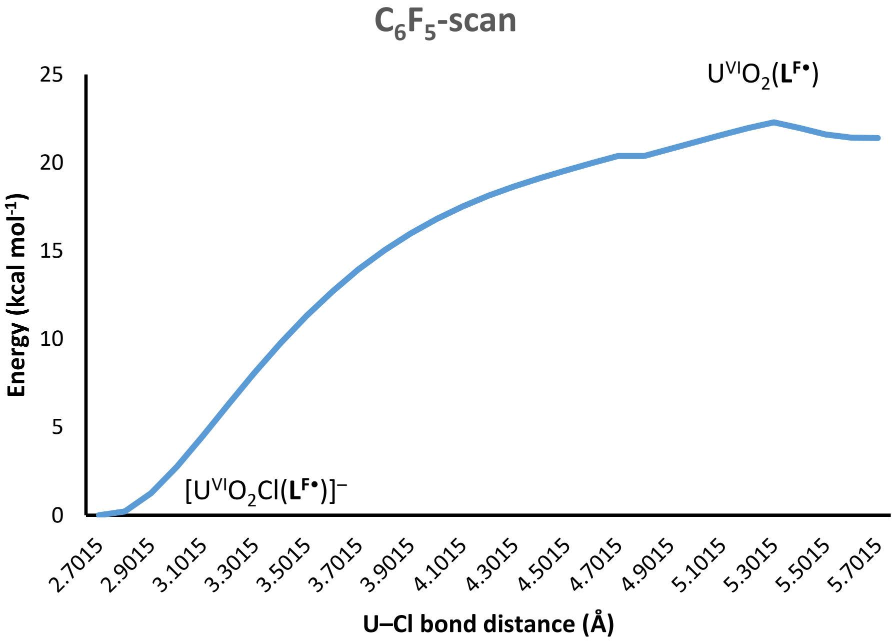


(b)

**Figure S6**: DFT scan for the U–Cl bond dissociation for (a) $[U^{VI}O_2Cl(\mathbf{L^{Mes}})]^-$ (b) $[U^{VI}O_2Cl(\mathbf{L^{F}})]^-$.

## 6.3 Optimized coordinates with dispersion correction

Compound $U^{VI}O_2Cl$(**L**$^{\mathbf{Mes}}$)

| | | | |
|---|---|---|---|
| U | 16.407733 | 4.473498 | 10.298825 |
| Cl | 17.833395 | 3.247486 | 8.425064 |
| O | 17.733400 | 5.253655 | 11.146597 |
| O | 15.025843 | 3.734197 | 9.515391 |
| N | 17.277730 | 2.371587 | 11.801343 |
| N | 15.095840 | 4.080203 | 12.394025 |
| N | 16.794907 | 6.611260 | 8.652525 |
| N | 14.830179 | 6.378962 | 10.681143 |
| C | 13.930550 | 4.589775 | 12.929517 |
| C | 15.396551 | 2.994584 | 13.130411 |
| C | 16.555633 | 2.192310 | 12.847563 |
| H | 16.791523 | 1.427024 | 13.587766 |
| C | 15.938645 | 7.544746 | 8.861238 |
| H | 15.975668 | 8.493018 | 8.323979 |
| C | 17.893118 | 6.867169 | 7.683232 |
| C | 10.784494 | 5.611924 | 12.704900 |
| C | 10.747381 | 4.706333 | 11.504786 |
| H | 11.172892 | 5.198310 | 10.623830 |
| H | 9.723341 | 4.407873 | 11.268971 |
| H | 11.338460 | 3.800224 | 11.674210 |
| C | 19.647755 | 2.482028 | 11.278986 |
| H | 19.439526 | 3.026645 | 10.358928 |
| H | 20.564567 | 1.901923 | 11.135614 |
| H | 19.812415 | 3.202405 | 12.085386 |
| C | 14.882441 | 7.418942 | 9.828538 |
| C | 13.269498 | 5.740016 | 12.484955 |
| C | 12.006112 | 6.114170 | 13.182655 |
| C | 13.698207 | 6.573279 | 11.446032 |
| C | 19.199742 | 6.433994 | 8.356063 |
| H | 19.374227 | 7.019811 | 9.263082 |
| H | 20.035071 | 6.597176 | 7.668161 |
| H | 19.174601 | 5.377548 | 8.620075 |
| C | 17.599749 | 6.035982 | 6.428744 |
| H | 17.489276 | 4.980501 | 6.675705 |
| H | 18.423471 | 6.143943 | 5.716119 |
| H | 16.679765 | 6.387554 | 5.950290 |
| C | 9.610596 | 5.976004 | 13.365485 |
| H | 8.661322 | 5.592666 | 12.997710 |
| C | 9.624169 | 6.817120 | 14.479683 |
| C | 10.853336 | 7.302691 | 14.929105 |
| H | 10.882605 | 7.963701 | 15.792562 |
| C | 12.051800 | 6.964670 | 14.298937 |
| C | 18.496133 | 1.535922 | 11.634582 |
| C | 18.890565 | 0.755969 | 12.895071 |
| H | 19.837193 | 0.246338 | 12.698546 |
| H | 18.163350 | -0.017653 | 13.163295 |
| H | 19.041800 | 1.417301 | 13.755114 |
| C | 18.214461 | 0.537439 | 10.505738 |
| H | 17.923374 | 1.053085 | 9.591062 |
| H | 17.411588 | -0.146747 | 10.799283 |
| H | 19.114516 | -0.051650 | 10.303554 |
| C | 18.030567 | 8.340004 | 7.277142 |
| H | 17.177078 | 8.704050 | 6.695548 |
| H | 18.912670 | 8.437016 | 6.639220 |
| H | 18.175116 | 8.992095 | 8.145347 |
| C | 13.798232 | 8.319975 | 10.026093 |
| H | 13.620757 | 9.232273 | 9.472574 |
| C | 14.446730 | 2.780096 | 14.168776 |
| H | 14.470240 | 1.980231 | 14.896675 |
| C | 13.513710 | 3.782941 | 14.036378 |
| H | 12.632967 | 3.964554 | 14.636211 |
| C | 13.364812 | 7.501268 | 14.799027 |
| H | 14.044173 | 6.688173 | 15.075881 |
| H | 13.220947 | 8.139604 | 15.673834 |
| H | 13.872271 | 8.088094 | 14.026076 |
| C | 13.043222 | 7.780848 | 11.042437 |
| H | 12.135220 | 8.168285 | 11.482853 |
| C | 8.348266 | 7.168743 | 15.194595 |
| H | 7.498843 | 7.196020 | 14.506076 |
| H | 8.420603 | 8.143193 | 15.685830 |
| H | 8.116469 | 6.428129 | 15.969546 |

Compound $[\mathrm{U^{VI}O_2Cl}(\mathbf{L}^{\mathrm{Mes}\bullet})]^-$

| | | | |
|---|---|---|---|
| U | 16.379875 | 4.491908 | 10.310471 |
| Cl | 17.854031 | 3.264659 | 8.359876 |
| O | 17.700828 | 5.242070 | 11.203797 |
| O | 15.035081 | 3.755846 | 9.451605 |
| N | 17.238595 | 2.347286 | 11.721253 |
| N | 15.046115 | 4.053141 | 12.347707 |
| N | 16.846032 | 6.613151 | 8.704491 |
| N | 14.812849 | 6.372302 | 10.676292 |
| C | 13.906315 | 4.568913 | 12.906572 |
| C | 15.358209 | 2.930228 | 13.070710 |
| C | 16.479423 | 2.130482 | 12.753556 |
| H | 16.684377 | 1.314888 | 13.449643 |
| C | 15.934619 | 7.527749 | 8.855936 |
| H | 15.953795 | 8.452574 | 8.275898 |
| C | 17.973041 | 6.898490 | 7.788416 |
| C | 10.762632 | 5.558235 | 12.789222 |
| C | 10.724471 | 4.573342 | 11.654349 |
| H | 11.178752 | 5.000332 | 10.754603 |
| H | 9.698270 | 4.272186 | 11.423621 |
| H | 11.306657 | 3.678549 | 11.898342 |
| C | 19.613119 | 2.441522 | 11.218277 |
| H | 19.409393 | 3.004369 | 10.307660 |
| H | 20.529014 | 1.857937 | 11.068849 |
| H | 19.774017 | 3.148610 | 12.037488 |
| C | 14.881097 | 7.417899 | 9.790943 |
| C | 13.241232 | 5.737793 | 12.474646 |
| C | 11.989329 | 6.120600 | 13.183186 |
| C | 13.683993 | 6.573849 | 11.426499 |
| C | 19.257152 | 6.419561 | 8.474737 |
| H | 19.403097 | 6.956261 | 9.416719 |
| H | 20.120138 | 6.603583 | 7.824262 |
| H | 19.205541 | 5.352094 | 8.687499 |
| C | 17.731760 | 6.143088 | 6.475481 |
| H | 17.608873 | 5.076918 | 6.668547 |
| H | 18.578515 | 6.285128 | 5.793403 |
| H | 16.826211 | 6.522179 | 5.988650 |
| C | 9.595040 | 5.929371 | 13.458853 |
| H | 8.646990 | 5.495252 | 13.146783 |
| C | 9.615233 | 6.844362 | 14.512772 |
| C | 10.841789 | 7.394218 | 14.888209 |
| H | 10.874000 | 8.112380 | 15.706138 |
| C | 12.028849 | 7.045093 | 14.240824 |
| C | 18.442410 | 1.506971 | 11.551114 |
| C | 18.833735 | 0.706088 | 12.804019 |
| H | 19.883960 | 0.206496 | 12.613172 |
| H | 18.106963 | -0.074770 | 13.052921 |
| H | 18.961724 | 1.357122 | 13.675793 |
| C | 18.180487 | 0.519107 | 10.407788 |
| H | 17.905822 | 1.055151 | 9.498853 |
| H | 17.363271 | -0.157524 | 10.681517 |
| H | 19.076665 | -0.080394 | 10.208360 |
| C | 18.150632 | 8.391692 | 7.466811 |
| H | 17.339072 | 8.795739 | 6.852230 |
| H | 19.076033 | 8.517291 | 6.896383 |
| H | 18.230897 | 8.991592 | 8.379885 |
| C | 13.796558 | 8.307230 | 9.978764 |
| H | 13.616298 | 9.214702 | 9.415184 |
| C | 14.422097 | 2.726742 | 14.111936 |
| H | 14.440335 | 1.916722 | 14.831089 |
| C | 13.501105 | 3.754038 | 14.010753 |
| H | 12.637238 | 3.943062 | 14.633914 |
| C | 13.342328 | 7.645718 | 14.657856 |
| H | 14.067036 | 6.860444 | 14.895889 |
| H | 13.223689 | 8.293457 | 15.531658 |
| H | 13.776676 | 8.232807 | 13.841740 |
| C | 13.033993 | 7.776827 | 11.003561 |
| H | 12.122231 | 8.170427 | 11.432698 |
| C | 8.350241 | 7.205331 | 15.245402 |
| H | 7.478432 | 7.158707 | 14.584862 |
| H | 8.404485 | 8.215588 | 15.663473 |
| H | 8.161939 | 6.517745 | 16.080086 |

Compound $U^{VI}O_2(\mathbf{L^{Mes\bullet}})$

| | | | |
|---|---|---|---|
| U | 16.562325 | 4.732652 | 10.633857 |
| O | 17.574508 | 5.649141 | 11.749331 |
| O | 15.741855 | 3.759366 | 9.413919 |
| N | 17.292367 | 2.616951 | 11.774016 |
| N | 15.029166 | 4.082719 | 12.369826 |
| N | 16.894572 | 6.534448 | 8.9151300 |
| N | 14.792211 | 6.359804 | 10.697698 |
| C | 13.863179 | 4.563843 | 12.901463 |
| C | 15.343837 | 2.936213 | 13.069272 |
| C | 16.504205 | 2.211955 | 12.739173 |
| H | 16.724614 | 1.306284 | 13.308974 |
| C | 15.957372 | 7.449770 | 8.890220 |
| H | 15.994024 | 8.279593 | 8.180617 |
| C | 18.020656 | 6.628318 | 7.971623 |
| C | 10.702705 | 5.601446 | 12.689635 |
| C | 10.667743 | 4.689048 | 11.494758 |
| H | 11.105762 | 5.175033 | 10.616499 |
| H | 9.643428 | 4.394991 | 11.251701 |
| H | 11.253758 | 3.781826 | 11.676334 |
| C | 19.177254 | 2.564768 | 10.291963 |
| H | 18.511099 | 2.608052 | 9.422076 |
| H | 20.090147 | 2.052939 | 9.975055 |
| H | 19.464031 | 3.578485 | 10.596017 |
| C | 14.871836 | 7.389743 | 9.783542 |
| C | 13.193623 | 5.729207 | 12.46891 |
| C | 11.928575 | 6.103421 | 13.158175 |
| C | 13.649322 | 6.561617 | 11.423485 |
| C | 18.922196 | 5.430395 | 8.265229 |
| H | 19.291779 | 5.474249 | 9.296641 |
| H | 19.796556 | 5.414520 | 7.608700 |
| H | 18.377765 | 4.492854 | 8.101166 |
| C | 17.513873 | 6.541973 | 6.527557 |
| H | 16.928217 | 5.628794 | 6.386176 |
| H | 18.353559 | 6.53281 | 5.825272 |
| H | 8.042782 | 6.444981 | 15.963816 |
| H | 16.877599 | 7.395794 | 6.2768800 |
| C | 9.526817 | 5.967035 | 13.346616 |
| H | 8.577035 | 5.581134 | 12.981638 |
| C | 9.540174 | 6.814724 | 14.455673 |
| C | 10.769402 | 7.303888 | 14.901076 |
| H | 10.798395 | 7.970273 | 15.760901 |
| C | 11.966424 | 6.961058 | 14.270440 |
| C | 18.495974 | 1.834587 | 11.448113 |
| C | 19.444383 | 1.790302 | 12.651505 |
| H | 20.374604 | 1.276221 | 12.389243 |
| H | 18.994086 | 1.257602 | 13.494214 |
| H | 19.685334 | 2.805358 | 12.980869 |
| C | 18.108101 | 0.421432 | 10.99843 |
| H | 17.40544 | 0.470339 | 10.161339 |
| H | 17.63266 | -0.138538 | 11.808999 |
| H | 18.994148 | -0.137446 | 10.680756 |
| C | 18.805576 | 7.924139 | 8.204909 |
| H | 18.195155 | 8.805115 | 7.986074 |
| H | 19.68841 | 7.960135 | 7.558661 |
| H | 19.131211 | 7.986697 | 9.247449 |
| C | 13.763641 | 8.260479 | 9.929131 |
| H | 13.574355 | 9.149768 | 9.342011 |
| C | 14.364023 | 2.683086 | 14.060923 |
| H | 14.361824 | 1.850518 | 14.752444 |
| C | 13.435096 | 3.699425 | 13.959604 |
| H | 12.541514 | 3.84764 | 14.550035 |
| C | 13.280847 | 7.499615 | 14.764096 |
| H | 13.962438 | 6.685623 | 15.032778 |
| H | 13.142505 | 8.137617 | 15.640782 |
| H | 13.782584 | 8.085158 | 13.98635 |
| C | 12.995603 | 7.746079 | 10.954624 |
| H | 12.069996 | 8.135963 | 11.355041 |
| C | 8.264387 | 7.169343 | 15.17039 |
| H | 7.410634 | 7.173547 | 14.486339 |
| H | 8.329520 | 8.155559 | 15.639346 |

Compound $U^{V}O_2$(**L**$^{\mathbf{Mes}}$)

| | | | | | | | |
|---|---|---|---|---|---|---|---|
| U | 8.561290 | 7.462548 | 1.295047 | C | 5.126269 | 10.279415 | 3.518578 |
| O | 7.331714 | 6.362694 | 0.345663 | H | 4.480860 | 9.8531650 | 2.746854 |
| O | 9.711340 | 8.520440 | 2.198558 | H | 4.950869 | 11.358307 | 3.551714 |
| N | 7.802311 | 5.924253 | 3.138668 | H | 4.828325 | 9.8714420 | 4.489022 |
| N | 9.885576 | 5.317102 | 1.181009 | C | 7.664484 | 1.4646190 | 2.170446 |
| N | 6.864135 | 8.559199 | 3.016856 | C | 8.287316 | -0.497256 | 3.478667 |
| N | 10.593109 | 7.482996 | -0.450693 | C | 10.202073 | 9.8261480 | -1.085882 |
| C | 6.252072 | 5.309418 | 4.711311 | H | 10.671842 | 10.716773 | -1.514802 |
| H | 5.457208 | 5.403422 | 5.439309 | H | 9.874681 | 10.064072 | -0.071316 |
| C | 10.835058 | 5.215650 | 0.235807 | H | 9.318594 | 9.576112 | 1.672275 |
| C | 9.651946 | 4.039968 | 1.637927 | C | 7.538453 | 0.106994 | 2.466890 |
| C | 7.859055 | 4.559565 | 3.313357 | H | 6.835336 | -0.492510 | 1.892528 |
| C | 11.214308 | 8.68472 | -1.070994 | C | 9.334356 | 1.655104 | 3.944841 |
| C | 8.709504 | 3.687697 | 2.616607 | C | 11.663348 | 8.402483 | -2.509104 |
| C | 6.463888 | 7.764949 | 3.945861 | H | 12.461464 | 7.656591 | -2.569301 |
| H | 5.844311 | 8.113437 | 4.777757 | H | 12.043996 | 9.323783 | -2.958838 |
| C | 6.832274 | 6.372402 | 3.954853 | H | 10.816219 | 8.057221 | -3.106502 |
| C | 11.258753 | 6.384322 | -0.494335 | C | 9.177331 | 0.293224 | 4.207570 |
| H | 12.188249 | 6.292046 | -1.064863 | H | 9.765201 | -0.159525 | 5.003204 |
| C | 6.597749 | 10.012526 | 3.18313 | C | 6.849012 | 2.099342 | 1.078435 |
| C | 6.948138 | 10.733713 | 1.884815 | H | 6.188705 | 1.369839 | 0.603533 |
| H | 8.001263 | 10.587058 | 1.634259 | H | 7.492475 | 2.535691 | 0.308512 |
| H | 6.769290 | 11.806283 | 2.007684 | H | 6.236785 | 2.918647 | 1.467115 |
| H | 6.351308 | 10.372515 | 1.047730 | C | 7.523522 | 10.511232 | 4.300884 |
| C | 10.513949 | 3.120609 | 0.949341 | H | 7.263496 | 10.057508 | 5.263023 |
| H | 10.541302 | 2.052394 | 1.114149 | H | 7.443783 | 11.598346 | 4.400256 |
| C | 6.892925 | 4.162900 | 4.299501 | H | 8.557809 | 10.249995 | 4.061325 |
| H | 6.724320 | 3.147578 | 4.630562 | C | 10.296918 | 2.490975 | 4.742670 |
| C | 12.402054 | 9.082334 | -0.183706 | H | 9.777706 | 3.305101 | 5.259374 |
| H | 13.182574 | 8.314383 | -0.195603 | H | 11.046115 | 2.956387 | 4.093510 |
| H | 12.059247 | 9.213728 | 0.846195 | H | 10.817571 | 1.887291 | 5.489854 |
| H | 12.838924 | 10.02174 | -0.536773 | C | 8.157860 | -1.970035 | 3.757079 |
| C | 11.268806 | 3.865630 | 0.071155 | H | 7.143295 | -2.32692 | 3.557171 |
| H | 12.023248 | 3.516584 | -0.621347 | H | 8.402351 | -2.203401 | 4.797273 |
| C | 8.570594 | 2.233683 | 2.919508 | H | 8.838494 | -2.550983 | 3.122872 |

Compound $[U^{V}O_2(\mathbf{L}^{\mathbf{Mes}})]_2$

| | | | |
|---|---|---|---|
| U | 8.561290 | 7.462548 | 1.295047 |
| O | 7.331714 | 6.362694 | 0.345663 |
| O | 9.711340 | 8.520440 | 2.198558 |
| N | 7.802311 | 5.924253 | 3.138668 |
| N | 9.885576 | 5.317102 | 1.181009 |
| N | 6.864135 | 8.559199 | 3.016856 |
| N | 10.593109 | 7.482996 | -0.450693 |
| C | 6.252072 | 5.309418 | 4.711311 |
| H | 5.457208 | 5.403422 | 5.439309 |
| C | 10.835058 | 5.215650 | 0.235807 |
| C | 9.651946 | 4.039968 | 1.637927 |
| C | 7.859055 | 4.559565 | 3.313357 |
| C | 11.214308 | 8.684720 | -1.070994 |
| C | 8.709504 | 3.687697 | 2.616607 |
| C | 6.463888 | 7.764949 | 3.945861 |
| H | 5.844311 | 8.113437 | 4.777757 |
| C | 6.832274 | 6.372402 | 3.954853 |
| C | 11.258753 | 6.384322 | -0.494335 |
| H | 12.188249 | 6.292046 | -1.064863 |
| C | 6.597749 | 10.012526 | 3.183130 |
| C | 6.948138 | 10.733713 | 1.884815 |
| H | 8.001263 | 10.587058 | 1.634259 |
| H | 6.769290 | 11.806283 | 2.007684 |
| H | 6.351308 | 10.372515 | 1.047730 |
| C | 10.513949 | 3.120609 | 0.949341 |
| H | 10.541302 | 2.052394 | 1.114149 |
| C | 6.892925 | 4.162900 | 4.299501 |
| H | 6.724320 | 3.147578 | 4.630562 |
| C | 12.402054 | 9.082334 | -0.183706 |
| H | 13.182574 | 8.314383 | -0.195603 |
| H | 12.059247 | 9.213728 | 0.846195 |
| H | 12.838924 | 10.021740 | -0.536773 |
| C | 11.268806 | 3.865630 | 0.071155 |
| H | 12.023248 | 3.516584 | -0.621347 |
| C | 5.126269 | 10.279415 | 3.518578 |
| H | 4.480860 | 9.853165 | 2.746854 |
| H | 4.950869 | 11.358307 | 3.551714 |
| H | 4.828325 | 9.871442 | 4.489022 |
| C | 7.664484 | 1.464619 | 2.170446 |
| C | 8.287316 | -0.497256 | 3.478667 |
| C | 10.202073 | 9.826148 | -1.085882 |
| H | 10.671842 | 10.716773 | -1.514802 |
| H | 9.874681 | 10.064072 | -0.071316 |
| H | 9.318594 | 9.576112 | -1.672275 |
| C | 7.538453 | 0.106994 | 2.466890 |
| H | 6.835336 | -0.492510 | 1.892528 |
| C | 9.334356 | 1.655104 | 3.944841 |
| C | 11.663348 | 8.402483 | -2.509104 |
| H | 12.461464 | 7.656591 | -2.569301 |
| H | 12.043996 | 9.323783 | -2.958838 |
| H | 10.816219 | 8.057221 | -3.106502 |
| C | 9.177331 | 0.293224 | 4.207570 |
| H | 9.765201 | -0.159525 | 5.003204 |
| C | 6.849012 | 2.099342 | 1.078435 |
| H | 6.188705 | 1.369839 | 0.603533 |
| C | 7.523522 | 10.511232 | 4.300884 |
| H | 7.263496 | 10.057508 | 5.263023 |
| H | 7.443783 | 11.598346 | 4.400256 |
| H | 8.557809 | 10.249995 | 4.061325 |
| C | 10.296918 | 2.490975 | 4.742670 |
| H | 9.777706 | 3.305101 | 5.259374 |
| H | 11.046115 | 2.956387 | 4.093510 |
| H | 10.817571 | 1.887291 | 5.489854 |
| C | 8.157860 | -1.970035 | 3.757079 |
| H | 7.143295 | -2.326920 | 3.557171 |
| H | 8.402351 | -2.203401 | 4.797273 |
| H | 8.838494 | -2.550983 | 3.122872 |
| C | 8.570594 | 2.233683 | 2.919508 |
| U | 6.292710 | 7.677819 | -1.294921 |
| O | 7.522202 | 8.777715 | -0.345476 |
| O | 5.142583 | 6.619938 | -2.198344 |
| N | 7.051519 | 9.216070 | -3.138658 |
| N | 4.968407 | 9.823300 | -1.180943 |
| N | 7.989673 | 6.581125 | -3.016792 |
| N | 4.260793 | 7.657310 | 0.450512 |
| C | 8.601680 | 9.830833 | -4.711416 |
| H | 9.396508 | 9.736790 | -5.439449 |
| C | 4.019011 | 9.924761 | -0.235673 |
| C | 5.202049 | 11.100433 | -1.637846 |
| C | 6.994773 | 10.580752 | -3.313414 |
| C | 3.639565 | 6.455644 | 1.070876 |
| C | 6.144418 | 11.452665 | -2.616611 |
| C | 8.389926 | 7.375349 | -3.945810 |
| H | 9.009510 | 7.026835 | -4.777693 |
| C | 8.021523 | 8.767891 | -3.954862 |
| C | 3.595283 | 8.756046 | 0.494392 |
| H | 2.665903 | 8.848376 | 1.065106 |
| C | 8.256095 | 5.127814 | -3.183030 |
| C | 7.905842 | 4.406671 | -1.884655 |
| H | 6.852703 | 4.553209 | -1.634085 |
| H | 8.084836 | 3.334115 | -2.007427 |
| H | 8.502638 | 4.768037 | -1.047621 |
| C | 4.340175 | 12.019819 | -0.949126 |
| H | 4.312864 | 13.088041 | -1.113891 |
| C | 7.960825 | 10.977366 | -4.299658 |
| H | 8.129397 | 11.992669 | -4.630794 |
| C | 2.451897 | 6.057947 | 0.183531 |
| H | 1.671381 | 6.825905 | 0.195271 |
| H | 2.794790 | 5.926429 | -0.846327 |
| H | 2.014984 | 5.118585 | 0.536664 |
| C | 3.585371 | 11.274802 | -0.070887 |
| H | 2.831022 | 11.623856 | 0.621712 |
| C | 9.727556 | 4.860992 | -3.518604 |
| H | 10.373018 | 5.287245 | -2.746926 |
| H | 9.902995 | 3.782107 | -3.551789 |
| H | 10.025399 | 5.269010 | -4.489062 |
| C | 7.189672 | 13.675676 | -2.170666 |
| C | 6.566728 | 15.637581 | -3.478788 |
| C | 4.651811 | 5.314223 | 1.085970 |
| H | 4.182000 | 4.423648 | 1.514948 |

| | | | |
|---|---|---|---|
| H | 7.492475 | 2.535691 | 0.308512 |
| H | 6.236785 | 2.918647 | 1.467115 |
| C | 7.315742 | 15.033289 | -2.467149 |
| H | 8.019014 | 15.632749 | -1.892931 |
| C | 5.519457 | 13.485288 | -3.944749 |
| C | 3.190409 | 6.738065 | 2.508913 |
| H | 2.392330 | 7.484013 | 2.568946 |
| H | 2.809665 | 5.816837 | 2.958714 |
| H | 4.037504 | 7.083342 | 3.106349 |
| C | 5.676524 | 14.847154 | -4.207518 |
| H | 5.088537 | 15.299935 | -5.003048 |
| C | 8.005316 | 13.040905 | -1.078810 |
| H | 8.665882 | 13.770332 | -0.604151 |
| H | 7.361969 | 12.604757 | -0.308678 |
| C | 6.283365 | 12.906668 | -2.919547 |
| H | 4.979282 | 5.076185 | 0.071458 |
| H | 5.535245 | 5.564315 | 1.672412 |
| H | 8.617289 | 12.221445 | -1.467566 |
| C | 7.330251 | 4.629035 | -4.300697 |
| H | 7.590174 | 5.082756 | -5.262865 |
| H | 7.410044 | 3.541923 | -4.400051 |
| H | 6.295973 | 4.890223 | -4.061050 |
| C | 4.556687 | 12.649474 | -4.742387 |
| H | 5.075745 | 11.835315 | -5.259193 |
| H | 3.807591 | 12.184108 | -4.093077 |
| H | 4.035921 | 13.253189 | -5.489467 |
| C | 6.696234 | 17.110348 | -3.757242 |
| H | 7.710839 | 17.467183 | -3.557449 |
| H | 6.451645 | 17.343706 | -4.797415 |
| H | 6.015697 | 17.691344 | -3.122976 |

Compound $U^{VI}O_2Cl(\mathbf{L^F})$

| | | | | | | | |
|---|---|---|---|---|---|---|---|
| C | 13.491925 | 2.837415 | 4.850693 | C | 10.277602 | 11.071919 | 3.722928 |
| H | 12.615384 | 2.351263 | 5.291105 | H | 10.145299 | 11.901334 | 3.041562 |
| H | 14.240417 | 2.069464 | 4.632208 | C | 11.234523 | 10.024925 | 3.593634 |
| H | 13.200013 | 3.313641 | 3.915274 | C | 12.208576 | 9.878710 | 2.545372 |
| C | 15.336905 | 4.529414 | 5.271601 | H | 12.312321 | 10.732295 | 1.874734 |
| H | 15.133575 | 5.018421 | 4.319852 | C | 13.971435 | 8.807399 | 1.359107 |
| H | 16.110101 | 3.771962 | 5.110707 | C | 14.288934 | 10.195335 | 0.787628 |
| H | 15.718409 | 5.273629 | 5.976635 | H | 13.460642 | 10.621399 | 0.211940 |
| C | 14.457121 | 3.134068 | 7.128136 | H | 15.132436 | 10.096369 | 0.099844 |
| H | 14.841550 | 3.822641 | 7.888410 | H | 14.579858 | 10.902441 | 1.571933 |
| H | 15.252156 | 2.420609 | 6.897589 | C | 13.461281 | 7.910963 | 0.224729 |
| H | 13.627104 | 2.559470 | 7.552805 | H | 13.217939 | 6.914485 | 0.591936 |
| C | 14.077221 | 3.859873 | 5.831860 | H | 14.233436 | 7.821230 | -0.545691 |
| C | 12.473249 | 5.022599 | 7.151773 | H | 12.567647 | 8.350321 | -0.230507 |
| H | 12.681034 | 4.329890 | 7.967583 | C | 15.249965 | 8.248078 | 1.991217 |
| C | 11.510339 | 6.050918 | 7.441685 | H | 15.579820 | 8.886677 | 2.815518 |
| C | 10.714520 | 6.155453 | 8.618055 | H | 16.042471 | 8.213135 | 1.237490 |
| H | 10.734291 | 5.479429 | 9.462048 | H | 15.093028 | 7.239103 | 2.370311 |
| C | 9.933313 | 7.272115 | 8.442852 | N | 13.058588 | 4.927426 | 6.013617 |
| H | 9.205203 | 7.683963 | 9.127043 | N | 11.262675 | 7.052310 | 6.579121 |
| C | 10.283060 | 7.824310 | 7.167567 | N | 11.152997 | 9.143330 | 4.605628 |
| C | 9.738253 | 8.985388 | 6.611428 | N | 12.928258 | 8.823515 | 2.420218 |
| C | 8.642822 | 9.641534 | 7.373496 | O | 10.837523 | 6.394378 | 3.770820 |
| C | 8.852439 | 10.842611 | 8.050704 | O | 13.897703 | 7.604214 | 4.978549 |
| C | 7.832719 | 11.459457 | 8.767630 | F | 10.052557 | 11.421204 | 8.028206 |
| C | 6.573127 | 10.868763 | 8.813291 | F | 8.053467 | 12.603474 | 9.411519 |
| C | 6.339022 | 9.669782 | 8.146112 | F | 5.592384 | 11.450702 | 9.495232 |
| C | 7.372613 | 9.068265 | 7.435697 | F | 5.131974 | 9.110847 | 8.189578 |
| C | 10.131728 | 9.593166 | 5.416281 | F | 7.124863 | 7.925022 | 6.799577 |
| C | 9.572620 | 10.794043 | 4.868825 | Cl | 13.413700 | 5.351764 | 2.511913 |
| H | 8.753191 | 11.356395 | 5.293169 | U | 12.389224 | 6.972389 | 4.340096 |

Compound $[U^{VI}O_2Cl(\mathbf{L^{F\bullet}})]^-$

| | | | | | | | |
|---|---|---|---|---|---|---|---|
| C | 13.423494 | 2.834534 | 4.773035 | C | 10.100876 | 10.910200 | 3.554380 |
| H | 12.528111 | 2.373153 | 5.203792 | H | 9.904600 | 11.682594 | 2.821059 |
| H | 14.146833 | 2.043548 | 4.543001 | C | 11.114042 | 9.926313 | 3.472687 |
| H | 13.149844 | 3.339441 | 3.846354 | C | 12.090147 | 9.772676 | 2.461652 |
| C | 15.310679 | 4.464754 | 5.219066 | H | 12.115791 | 10.559709 | 1.704740 |
| H | 15.114398 | 4.981877 | 4.280141 | C | 14.031387 | 8.833672 | 1.446634 |
| H | 16.063531 | 3.689005 | 5.037563 | C | 14.366098 | 10.259651 | 0.976277 |
| H | 15.712377 | 5.184572 | 5.938797 | H | 13.584196 | 10.699648 | 0.348587 |
| C | 14.395368 | 3.067020 | 7.050110 | H | 15.277192 | 10.225048 | 0.371025 |
| H | 14.807298 | 3.729722 | 7.819200 | H | 14.546736 | 10.924639 | 1.827578 |
| H | 15.162957 | 2.328435 | 6.800562 | C | 13.615982 | 7.996282 | 0.231060 |
| H | 13.545832 | 2.520634 | 7.474328 | H | 13.378297 | 6.976930 | 0.537264 |
| C | 14.026984 | 3.831726 | 5.769776 | H | 14.427611 | 7.967200 | -0.505489 |
| C | 12.484183 | 5.040300 | 7.132441 | H | 12.732934 | 8.438618 | -0.243823 |
| H | 12.696835 | 4.339151 | 7.941228 | C | 15.288925 | 8.261669 | 2.108702 |
| C | 11.569641 | 6.070675 | 7.455621 | H | 15.561316 | 8.860887 | 2.982146 |
| C | 10.854965 | 6.227561 | 8.665559 | H | 16.122366 | 8.272968 | 1.396947 |
| H | 10.927647 | 5.584716 | 9.534145 | H | 15.121956 | 7.234440 | 2.431561 |
| C | 10.059215 | 7.345555 | 8.500207 | N | 13.045196 | 4.920096 | 5.968607 |
| H | 9.383753 | 7.786195 | 9.220951 | N | 11.245138 | 7.060654 | 6.565667 |
| C | 10.312402 | 7.846660 | 7.185364 | N | 11.091336 | 9.108687 | 4.573448 |
| C | 9.712023 | 8.993152 | 6.611337 | N | 12.940242 | 8.790393 | 2.448653 |
| C | 8.644546 | 9.658328 | 7.383006 | O | 10.853373 | 6.362432 | 3.733170 |
| C | 8.791533 | 10.959693 | 7.876427 | O | 13.869739 | 7.624496 | 5.029343 |
| C | 7.787931 | 11.596119 | 8.598679 | F | 9.927559 | 11.630740 | 7.677756 |
| C | 6.599138 | 10.926132 | 8.863617 | F | 7.964027 | 12.839979 | 9.062004 |
| C | 6.422482 | 9.628554 | 8.396871 | F | 5.630559 | 11.526576 | 9.564051 |
| C | 7.432659 | 9.016192 | 7.662990 | F | 5.271760 | 8.989271 | 8.640399 |
| C | 10.066993 | 9.554999 | 5.362985 | F | 7.204742 | 7.783017 | 7.209926 |
| C | 9.432295 | 10.676077 | 4.740176 | Cl | 13.452341 | 5.366424 | 2.450386 |
| H | 8.586925 | 11.220979 | 5.137910 | U | 12.369217 | 6.991280 | 4.358241 |

Compound $U^{VI}O_2(\mathbf{L^{F\bullet}})$

| | | | | | | | |
|---|---|---|---|---|---|---|---|
| C | 13.423494 | 2.834534 | 4.773035 | C | 10.100876 | 10.910200 | 3.554380 |
| H | 12.528111 | 2.373153 | 5.203792 | H | 9.904600 | 11.682594 | 2.821059 |
| H | 14.146833 | 2.043548 | 4.543001 | C | 11.114042 | 9.926313 | 3.472687 |
| H | 13.149844 | 3.339441 | 3.846354 | C | 12.090147 | 9.772676 | 2.461652 |
| C | 15.310679 | 4.464754 | 5.219066 | H | 12.115791 | 10.559709 | 1.704740 |
| H | 15.114398 | 4.981877 | 4.280141 | C | 14.031387 | 8.833672 | 1.446634 |
| H | 16.063531 | 3.689005 | 5.037563 | C | 14.366098 | 10.259651 | 0.976277 |
| H | 15.712377 | 5.184572 | 5.938797 | H | 13.584196 | 10.699648 | 0.348587 |
| C | 14.395368 | 3.067020 | 7.050110 | H | 15.277192 | 10.225048 | 0.371025 |
| H | 14.807298 | 3.729722 | 7.819200 | H | 14.546736 | 10.924639 | 1.827578 |
| H | 15.162957 | 2.328435 | 6.800562 | C | 13.615982 | 7.996282 | 0.231060 |
| H | 13.545832 | 2.520634 | 7.474328 | H | 13.378297 | 6.976930 | 0.537264 |
| C | 14.026984 | 3.831726 | 5.769776 | H | 14.427611 | 7.967200 | -0.505489 |
| C | 12.484183 | 5.040300 | 7.132441 | H | 12.732934 | 8.438618 | -0.243823 |
| H | 12.696835 | 4.339151 | 7.941228 | C | 15.288925 | 8.261669 | 2.108702 |
| C | 11.569641 | 6.070675 | 7.455621 | H | 15.561316 | 8.860887 | 2.982146 |
| C | 10.854965 | 6.227561 | 8.665559 | H | 16.122366 | 8.272968 | 1.396947 |
| H | 10.927647 | 5.584716 | 9.534145 | H | 15.121956 | 7.23444 | 2.431561 |
| C | 10.059215 | 7.345555 | 8.500207 | N | 13.045196 | 4.920096 | 5.968607 |
| H | 9.383753 | 7.786195 | 9.220951 | N | 11.245138 | 7.060654 | 6.565667 |
| C | 10.312402 | 7.846660 | 7.185364 | N | 11.091336 | 9.108687 | 4.573448 |
| C | 9.712023 | 8.993152 | 6.611337 | N | 12.940242 | 8.790393 | 2.448653 |
| C | 8.644546 | 9.65832 0 | 7.383006 | O | 10.853373 | 6.362432 | 3.733170 |
| C | 8.791533 | 10.959693 | 7.876427 | O | 13.869739 | 7.624496 | 5.029343 |
| C | 7.787931 | 11.596119 | 8.598679 | F | 9.927559 | 11.63074 | 7.677756 |
| C | 6.599138 | 10.926132 | 8.863617 | F | 7.964027 | 12.839979 | 9.062004 |
| C | 6.422482 | 9.628554 | 8.396871 | F | 5.630559 | 11.526576 | 9.564051 |
| C | 7.432659 | 9.016192 | 7.662990 | F | 5.271760 | 8.989271 | 8.640399 |
| C | 10.066993 | 9.554999 | 5.362985 | F | 7.204742 | 7.783017 | 7.209926 |
| C | 9.432295 | 10.676077 | 4.740176 | Cl | 13.452341 | 5.366424 | 2.450386 |
| H | 8.586925 | 11.220979 | 5.137910 | U | 12.369217 | 6.99128 | 4.358241 |

Compound $U^{V}O_2(\mathbf{L}^{\mathbf{F}})$

| | | | |
|---|---|---|---|
| U | 6.27298 | 7.605653 | 1.286915 |
| O | 7.423705 | 8.775915 | 0.335364 |
| O | 5.169751 | 6.505209 | 2.19027 |
| N | 7.064877 | 9.128405 | 3.123634 |
| N | 4.918840 | 9.728190 | 1.232801 |
| N | 8.014304 | 6.507173 | 2.966174 |
| N | 4.245085 | 7.569076 | 0.407777 |
| C | 8.595743 | 9.729047 | -4.718434 |
| H | 9.378418 | 9.629700 | -5.458453 |
| C | 3.966915 | 9.824448 | -0.288935 |
| C | 5.188815 | 11.012831 | -1.647194 |
| C | 7.012798 | 10.491365 | -3.29918 |
| C | 3.672878 | 6.359249 0 | 1.058575 |
| C | 6.162761 | 11.356431 | -2.594889 |
| C | 8.393689 | 7.280941 | -3.921604 |
| H | 8.998688 | 6.917135 | -4.757185 |
| C | 8.021928 | 8.672291 | -3.948992 |
| C | 3.555332 | 8.654703 | 0.444349 |
| H | 2.630663 | 8.734312 | 1.023137 |
| C | 8.298137 | 5.052433 | -3.105261 |
| C | 7.954749 | 4.347300 | -1.796393 |
| H | 6.896762 | 4.473088 | -1.555301 |
| H | 8.156771 | 3.276730 | -1.896831 |
| H | 8.537413 | 4.737471 | -0.963057 |
| C | 4.341298 | 11.93129 | -0.936936 |
| H | 4.339475 | 13.006132 | -1.051325 |
| C | 7.967986 | 10.879415 | -4.30026 |
| H | 8.137284 | 11.887055 | -4.653412 |
| C | 2.504318 | 5.889673 | 0.181671 |
| H | 1.694966 | 6.627320 | 0.169473 |
| H | 2.853277 | 5.738961 | -0.843425 |
| H | 2.103397 | 4.9454870 | 0.563194 |
| C | 3.559666 | 11.179086 | -0.092154 |
| H | 2.810148 | 11.526621 | 0.606042 |
| C | 9.775953 | 4.8055170 | -3.429995 |
| H | 10.410616 | 5.2569570 | -2.663422 |
| H | 9.970387 | 3.7295190 | -3.443048 |
| H | 10.070435 | 5.2019630 | -4.406192 |
| C | 7.471861 | 13.469785 | -2.328921 |
| C | 6.751309 | 15.550067 | -3.292693 |
| C | 4.737794 | 5.2677090 | 1.104214 |
| H | 4.322030 | 4.3671800 | 1.564512 |
| H | 5.071458 | 5.010519 | 0.096205 |
| C | 7.676731 | 14.828323 | -2.544227 |
| C | 5.444807 | 13.554479 | -3.591715 |
| C | 3.209448 | 6.662812 | 2.487694 |
| H | 2.367262 | 7.360152 | 2.525161 |
| H | 2.885991 | 5.735610 | 2.968566 |
| H | 4.035003 | 7.078679 | 3.070646 |
| C | 5.630677 | 14.914487 | -3.818981 |
| C | 7.386108 | 4.518855 | -4.218198 |
| H | 7.642183 | 4.957239 | -5.188355 |
| H | 7.484565 | 3.431652 | -4.295967 |
| H | 6.346140 | 4.765596 | -3.988220 |
| C | 6.356978 | 12.810003 | -2.846505 |
| F | 8.744777 | 15.442378 | -2.038192 |
| F | 8.364869 | 12.802437 | -1.598310 |
| F | 6.937819 | 16.849634 | -3.503888 |
| F | 4.749804 | 15.608793 | -4.537200 |
| F | 4.367861 | 12.965158 | -4.110427 |
| H | 5.573330837 | 5.639084677 | 1.67898204 |

Compound $[U^{V}O_2(\mathbf{L^F})]_2$

| | | | |
|---|---|---|---|
| U | 8.581025 | 7.534748 | 1.287048 |
| O | 7.430305 | 6.364495 | 0.335482 |
| O | 9.684324 | 8.635167 | 2.190344 |
| N | 7.789114 | 6.012005 | 3.123796 |
| N | 9.93512 | 5.412179 | 1.232917 |
| N | 6.839688 | 8.633254 | 2.966378 |
| N | 10.60885 | 7.571240 | -0.407739 |
| C | 6.258220 | 5.411366 | 4.718573 |
| H | 5.475560 | 5.510720 | 5.458608 |
| C | 10.88703 | 5.315890 | 0.289041 |
| C | 9.665103 | 4.127543 | 1.647300 |
| C | 7.841115 | 4.649033 | 3.299273 |
| C | 11.181053 | 8.780992 | -1.058675 |
| C | 8.691116 | 3.783959 | 2.594957 |
| C | 6.460381 | 7.859495 | 3.921848 |
| H | 5.855462 | 8.223313 | 4.757480 |
| C | 6.832088 | 6.468129 | 3.949178 |
| C | 11.298593 | 6.485607 | -0.444296 |
| H | 12.223228 | 6.405966 | -1.023134 |
| C | 6.555939 | 10.088012 | 3.105459 |
| C | 6.899370 | 10.793101 | 1.796580 |
| H | 7.957353 | 10.667253 | 1.555501 |
| H | 6.697399 | 11.863683 | 1.896992 |
| H | 6.316692 | 10.402938 | 0.963248 |
| C | 10.512602 | 3.209061 | 0.937054 |
| H | 10.514401 | 2.134220 | 1.051455 |
| C | 6.885886 | 4.260980 | 4.300314 |
| H | 6.716522 | 3.253328 | 4.653402 |
| C | 12.349743 | 9.250555 | -0.181937 |
| H | 13.159053 | 8.512860 | -0.169781 |
| H | 12.000912 | 9.401359 | 0.843189 |
| H | 12.750677 | 10.194691 | -0.563571 |
| C | 11.294249 | 3.961242 | 0.092266 |
| H | 12.043755 | 3.613686 | -0.605933 |
| C | 5.078143 | 10.335056 | 3.430192 |
| H | 4.443443 | 9.883640 | 2.663637 |
| H | 4.883795 | 11.41107 | 3.443208 |
| H | 4.783629 | 9.938672 | 4.406403 |
| C | 7.381920 | 1.670727 | 2.328632 |
| C | 8.102162 | -0.409681 | 3.292362 |
| C | 10.116202 | 9.872595 | -1.104278 |
| H | 10.531988 | 10.773077 | -1.564646 |
| H | 9.782624 | 10.129849 | -0.096257 |
| H | 9.243021 | 9.561385 | -1.676354 |
| C | 7.176913 | 0.312188 | 2.543801 |
| C | 9.408765 | 1.585786 | 3.591749 |
| C | 11.644304 | 8.477283 | -2.487822 |
| H | 12.486435 | 7.779878 | -2.525323 |
| H | 11.967774 | 9.404422 | -2.968804 |
| H | 10.818655 | 8.061426 | -3.070650 |
| C | 9.222756 | 0.225774 | 3.818880 |
| C | 7.468015 | 10.621539 | 4.218381 |
| H | 7.211922 | 10.183174 | 5.188541 |
| H | 7.36963 | 11.708748 | 4.296145 |
| H | 8.507964 | 10.374729 | 3.988394 |

| | | | |
|---|---|---|---|
| N | 4.245085 | 7.569076 | 0.407777 |
| C | 8.595743 | 9.729047 | -4.718434 |
| H | 9.378418 | 9.629700 | -5.458453 |
| C | 3.966915 | 9.824448 | -0.288935 |
| C | 5.188815 | 11.012831 | -1.647194 |
| C | 7.012798 | 10.491365 | -3.29918 |
| C | 3.672878 | 6.359249 | 1.058575 |
| C | 6.162761 | 11.356431 | -2.594889 |
| C | 8.393689 | 7.280941 | -3.921604 |
| H | 8.998688 | 6.917135 | -4.757185 |
| C | 8.021928 | 8.672291 | -3.948992 |
| C | 3.555332 | 8.654703 | 0.444349 |
| H | 2.630663 | 8.734312 | 1.023137 |
| C | 8.298137 | 5.052433 | -3.105261 |
| C | 7.954749 | 4.347300 | -1.796393 |
| H | 6.896762 | 4.473088 | -1.555301 |
| H | 8.156771 | 3.276730 | -1.896831 |
| H | 8.537413 | 4.737471 | -0.963057 |
| C | 4.341298 | 11.93129 | -0.936936 |
| H | 4.339475 | 13.006132 | -1.051325 |
| C | 7.967986 | 10.879415 | -4.300260 |
| H | 8.137284 | 11.887055 | -4.653412 |
| C | 2.504318 | 5.889673 | 0.181671 |
| H | 1.694966 | 6.627320 | 0.169473 |
| H | 2.853277 | 5.738961 | -0.843425 |
| H | 2.103397 | 4.945487 | 0.563194 |
| C | 3.559666 | 11.179086 | -0.092154 |
| H | 2.810148 | 11.526621 | 0.606042 |
| C | 9.775953 | 4.805517 | -3.429995 |
| H | 10.410616 | 5.256957 | -2.663422 |
| H | 9.970387 | 3.729519 | -3.443048 |
| H | 10.070435 | 5.201963 | -4.406192 |
| C | 7.471861 | 13.469785 | -2.328921 |
| C | 6.751309 | 15.550067 | -3.292693 |
| C | 4.737794 | 5.267709 | 1.104214 |
| H | 4.322030 | 4.367180 | 1.564512 |
| H | 5.071458 | 5.010519 | 0.096205 |
| H | 5.610918 | 5.578948 | 1.676360 |
| C | 7.676731 | 14.828323 | -2.544227 |
| C | 5.444807 | 13.554479 | -3.591715 |
| C | 3.209448 | 6.662812 | 2.487694 |
| H | 2.367262 | 7.360152 | 2.525161 |
| H | 2.885991 | 5.735610 | 2.968566 |
| H | 4.035003 | 7.078679 | 3.070646 |
| C | 5.630677 | 14.914487 | -3.818981 |
| C | 7.386108 | 4.518855 | -4.218198 |
| H | 7.642183 | 4.957239 | -5.188355 |
| H | 7.484565 | 3.431652 | -4.295967 |
| H | 6.34614 | 4.765596 | -3.988220 |
| C | 6.356978 | 12.810003 | -2.846505 |
| F | 10.485676 | 2.174990 | 4.110666 |
| F | 10.103465 | -0.468654 | 4.537183 |
| F | 7.915520 | -1.709251 | 3.503427 |
| F | 6.108901 | -0.301749 | 2.037550 |
| F | 6.489082 | 2.338207 | 1.597934 |

| | | | | | | | |
|---|---|---|---|---|---|---|---|
| C | 8.496768 | 2.330382 | 2.846451 | F | 8.744777 | 15.442378 | -2.038192 |
| U | 6.272980 | 7.605653 | -1.286915 | F | 8.364869 | 12.802437 | -1.59831 |
| O | 7.423705 | 8.775915 | -0.335364 | F | 6.937819 | 16.849634 | -3.503888 |
| O | 5.169751 | 6.505209 | -2.190270 | F | 4.749804 | 15.608793 | -4.53720 |
| N | 7.064877 | 9.128405 | -3.123634 | F | 4.367861 | 12.965158 | -4.110427 |
| N | 4.918840 | 9.728190 | -1.232801 | N | 8.014304 | 6.507173 | -2.966174 |

# 7 References


1. Sheldrick, G. M., *Acta Crystallogr., Sect. A: Found. Adv.* **2015,** *71*, 3−8.
2. Sheldrick, G. M., *Acta Crystallogr., Sect. C: Chem.* **2015,** *71*, 3−8.
3. Sheldrick, G. M., *Acta Crystallogr., Sect. A: Found. Adv.* **2008,** (64), 122.
4. O. V. Dolomanov, L. J. B., R. J. Gildea, J. A. K. Howard, H. Puschmann, *J. Appl. Crystallogr.* **2009,** *42*, 339−341.
5. Becke, A. D., *J. Chem. Phys* **1993,** *98*, 5648.
6. Burke, K.; Perdew, J. P.; Yang, W., *Electronic Density Functional Theory: Recent Progress and New Directions*. Plenum: New york, 1998.
7. Moritz, A.; Cao, X.; Dolg, M., *Theor. Chem. Acc.* **2007,** *118*, 845.
8. Hollwarth, A.; Bohme, M.; Dapprich, S.; Ehlers, A. W.; Gobbi, A.; Jonas, V.; Kohler, K. F.; Stegmann, R.; Veldkamp, A.; Frenkling, G., *J. Chem. Phys.* **1993,** *208*, 237.
9. Hariharan, P. C.; Pople, J. A., *Theor. Chim. Acta.* **1973,** *28*, 213.
10. Hehre, W. J.; Ditchfield, R.; Pople, J. A., *J. Chem. Phys.* **1972,** *56*, 2257.
11. Grimme, S.; Ehrlich, S.; Goerigk, L., *J. Comp. Chem.* **2011,** *32*, 1456.
12. Frisch, M. J.; Trucks, G. W.; Schlegel, H. B.; Scuseria, G. E.; Robb, M. A.; Cheeseman, J. R.; Scalmani, G.; Barone, V.; Petersson, G. A.; Nakatsuji, H.; Li, X.; Caricato, M.; Marenich, A. V.; Bloino, J.; Janesko, B. G.; Gomperts, R.; Mennucci, B.; Hratchian, H. P.; Ortiz, J. V.; Izmaylov, A. F.; Sonnenberg, J. L.; Williams; Ding, F.; Lipparini, F.; Egidi, F.; Goings, J.; Peng, B.; Petrone, A.; Henderson, T.; Ranasinghe, D.; Zakrzewski, V. G.; Gao, J.; Rega, N.; Zheng, G.; Liang, W.; Hada, M.; Ehara, M.; Toyota, K.; Fukuda, R.; Hasegawa, J.; Ishida, M.; Nakajima, T.; Honda, Y.; Kitao, O.; Nakai, H.; Vreven, T.; Throssell, K.; Montgomery Jr., J. A.; Peralta, J. E.; Ogliaro, F.; Bearpark, M. J.; Heyd, J. J.; Brothers, E. N.; Kudin, K. N.; Staroverov, V. N.; Keith, T. A.; Kobayashi, R.; Normand, J.; Raghavachari, K.; Rendell, A. P.; Burant, J. C.; Iyengar, S. S.; Tomasi, J.; Cossi, M.; Millam, J. M.; Klene, M.; Adamo, C.; Cammi, R.; Ochterski, J. W.; Martin, R. L.; Morokuma, K.; Farkas, O.; Foresman, J. B.; Fox, D. J. *Gaussian 16 Rev. C.01*, Wallingford, CT, 2016.
